# The Nencki-Symfonia electroencephalography/event-related potential dataset: Multiple cognitive tasks and resting-state data collected in a sample of healthy adults

**DOI:** 10.1093/gigascience/giac015

**Published:** 2022-03-07

**Authors:** Patrycja Dzianok, Ingrida Antonova, Jakub Wojciechowski, Joanna Dreszer, Ewa Kublik

**Affiliations:** Laboratory of Emotions Neurobiology, Nencki Institute of Experimental Biology PAS, 02-093, Warsaw, Poland; Laboratory of Neuroinformatics, Nencki Institute of Experimental Biology PAS, 02-093, Warsaw, Poland; Laboratory of Emotions Neurobiology, Nencki Institute of Experimental Biology PAS, 02-093, Warsaw, Poland; Bioimaging Research Center, Institute of Physiology and Pathology of Hearing, 02-042, Warsaw, Poland; Institute of Psychology, Faculty of Philosophy and Social Sciences, Nicolaus Copernicus University in Toruń, 87-100, Toruń, Poland; Laboratory of Emotions Neurobiology, Nencki Institute of Experimental Biology PAS, 02-093, Warsaw, Poland

## Abstract

**Background:**

One of the goals of neuropsychology is to understand the brain mechanisms underlying aspects of attention and cognitive control. Several tasks have been developed as a part of this body of research, however their results are not always consistent. A reliable comparison of the data and a synthesis of study conclusions has been precluded by multiple methodological differences. Here, we describe a publicly available, high-density electroencephalography (EEG) dataset obtained from 42 healthy young adults while they performed 3 cognitive tasks: (i) an extended multi-source interference task; (ii) a 3-stimuli oddball task; (iii) a control, simple reaction task; and (iv) a resting-state protocol. Demographic and psychometric information are included within the dataset.

**Dataset Validation:**

First, data validation confirmed acceptable quality of the obtained EEG signals. Typical event-related potential (ERP) waveforms were obtained, as expected for attention and cognitive control tasks (i.e., N200, P300, N450). Behavioral results showed the expected progression of reaction times and error rates, which confirmed the effectiveness of the applied paradigms.

**Conclusions:**

This dataset is well suited for neuropsychological research regarding common and distinct mechanisms involved in different cognitive tasks. Using this dataset, researchers can compare a wide range of classical EEG/ERP features across tasks for any selected subset of electrodes. At the same time, 128-channel EEG recording allows for source localization and detailed connectivity studies. Neurophysiological measures can be correlated with additional psychometric data obtained from the same participants. This dataset can also be used to develop and verify novel analytical and classification approaches that can advance the field of deep/machine learning algorithms, recognition of single-trial ERP responses to different task conditions, and detection of EEG/ERP features for use in brain-computer interface applications.

## Data Description

### Background and purpose

Here, we describe a dataset of electroencephalography (EEG)/event-related potential (ERP) signals and metadata that may be useful in ≥3 domains: (i) in-depth neuroscience investigations related to attention and cognitive control; (ii) testing machine and deep learning algorithms suited for neuroimage data, with a particular emphasis on attention and cognitive control aspects; and (iii) new approaches for brain-computer interface (BCI) development.

There is a tremendous need for neuroscience datasets to be available to the wider scientific community, which may help to overcome the reproducibility crisis and improve the quality of research (e.g., larger sample sizes, more reproducible findings). Open access to data may also increase the speed of research and reduce the need to collect multiple redundant datasets, such as in computational neuroscience [1–4].

**Figure 1:**
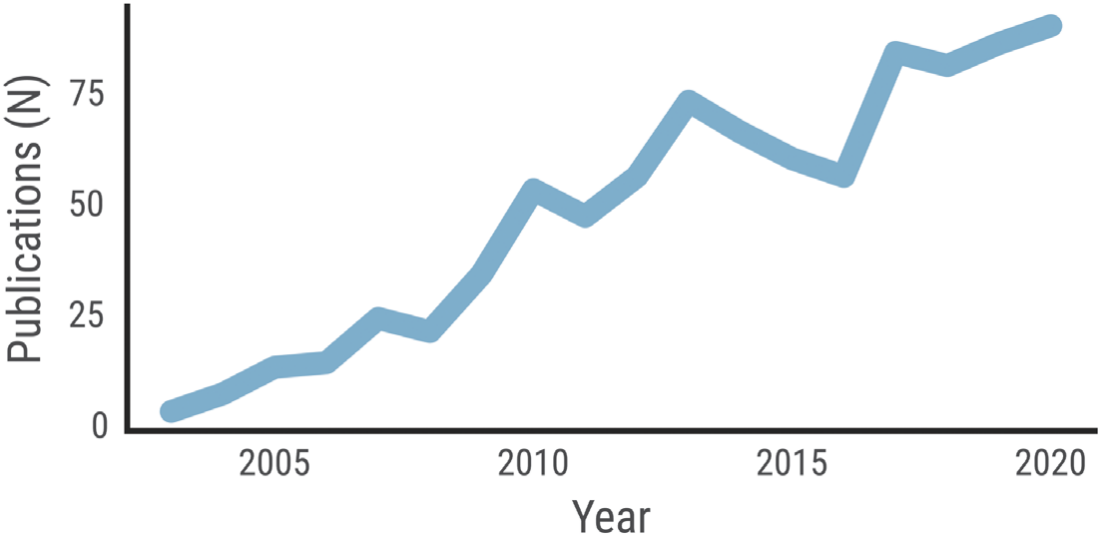
: Number of publications per year that use the multi-source interference task (MSIT), over the past 17 years. This summary includes both original and theoretical works with comments addressing MSIT published in Google Scholar using the keyword “multi-source interference task.” Of note, patents are excluded, and citations are included. In total, 868 papers were mentioning MSIT as of 16 August 2021.

Research concerning conflict processing and attentional cognitive control is well established in the neuroscience literature. Such research has focused primarily on characterizing conflict processing and attentional cognitive control using behavioral [5, 6, 7] and neuroimaging [8, 9, 10] approaches. This work has demonstrated 2 primary sources of cognitive conflict: (i) stimulus-stimulus interference, which arises from flanking stimuli that are similar to each other but differ from the target stimulus; and (ii) stimulus-response interference, which arises from spatial (in)compatibility between the target stimulus and the response button positions. Both sources of conflict may be examined using different sensory domains, also tactile and auditory ones [11]. The most common tasks used to study these conflict types include the Flanker task, which evokes the “Flanker effect” [12], and the Simon task, which evokes the “Simon effect” [13]. Both the Flanker and Simon effects are included in the multi-source interference task (MSIT) [14, 15], which was designed to maximize conflict effects and strongly engage conflict-specific brain areas, including the dorsal anterior cingulate cortex. The popularity of the MSIT task has increased rapidly over the past 17 years (Fig. 1).

There are still open questions related to conflict processing studies, including (i) the exact definition of conflict, (ii) how conflict influences reaction times, and (iii) how conflict is resolved by brain mechanisms. Furthermore, it is unclear whether the global phenomenon of “conflict” actually exists. If yes, it is also unclear whether conflict is resolved by a single, dedicated brain mechanism, regardless of the specific details of the conflicting events. In contrast, conflict may be detected and resolved by different neural mechanisms, depending on the type of conflicting stimuli. A reliable comparison of the data and a synthesis of study conclusions is precluded by multiple methodological differences. To our knowledge, only 2 MSIT-based datasets are publicly available: (i) a dataset that includes intracranial EEG with and without brain stimulation collected from 21 patients with epilepsy [16, 17] and (ii) a dataset of EEG/deep brain stimulation with affective MSIT collected from 14 patients with obsessive-compulsive disorder and 14 patients with major depressive disorder [18, 19]. However, these datasets are relatively limited in sample size (*N* < 25). To our knowledge, there are no publicly available MSIT datasets collected in healthy individuals. This precludes the ability to resolve and clarify ongoing and past studies.

Here, we describe an extended MSIT (MSIT+) dataset collected in healthy individuals. This database is well suited to address the aforementioned questions because it includes an MSIT+ task. In addition to non-conflict and multi-source conflict conditions, our task includes 2 single-conflict conditions (i.e., Simon and Flanker). The same participants performed the classical 3-stimuli visual oddball task with 2 rare stimuli: a target and a distractor. A simple reaction time task (SRT) was also included as a no-conflict, no-attention, control situation. Prior to completing tasks, EEG data were recorded during a 10 min resting-state (REST) condition. Resting-state paradigms are commonly used in neuroimaging and neurophysiology research to examine spontaneous functional activity of the brain at an individual level.

The included tasks initiate multiple cognitive processes, including attention, attentional control, working memory, and action selection. These tasks are of interest from the perspective of basic science and are also helpful for understanding how their dysfunctions induce various mental and medical conditions. The selected tasks are well suited to answer many related questions regarding the brain processes underlying conflict and attentional control. Indeed, we selected 2 attentional tasks (i.e., MSIT+ and oddball) and the SRT task, which provides information regarding processing speed of each participant regardless of attentional resources and effort/conflict. The additional REST procedure is well suited to evaluate task-independent (i.e., spontaneous) activity of the brain, while psychometric questionnaires allow correlation of EEG/ERP results with participants' individual characteristics. An in-depth analysis of such a database (i.e., same participants, same experimental conditions, *N* = 42) may also lead to new clinical applications.

### Experimental design

#### Participants and psychometric measures

Forty-two healthy, right-handed young adults (aged 20–34 years, 22 females (Table 1)) completed the described experiment. The experimental group was highly homogeneous in regard to the education and social status—the majority of the participants were students. Participants were provided with detailed information about the study and a list of exclusionary criteria, including contraindications for EEG/ERP: pregnancy, chronic diseases (e.g., epilepsy, chronic headaches, chronic sleep disorders), skin diseases and allergies (especially head-related), diagnosed mental and neurodevelopmental disorders, head injury, medication use (particularly those that may influence nervous system functioning), alcohol use, and psychoactive substance abuse.

**Figure 4:**
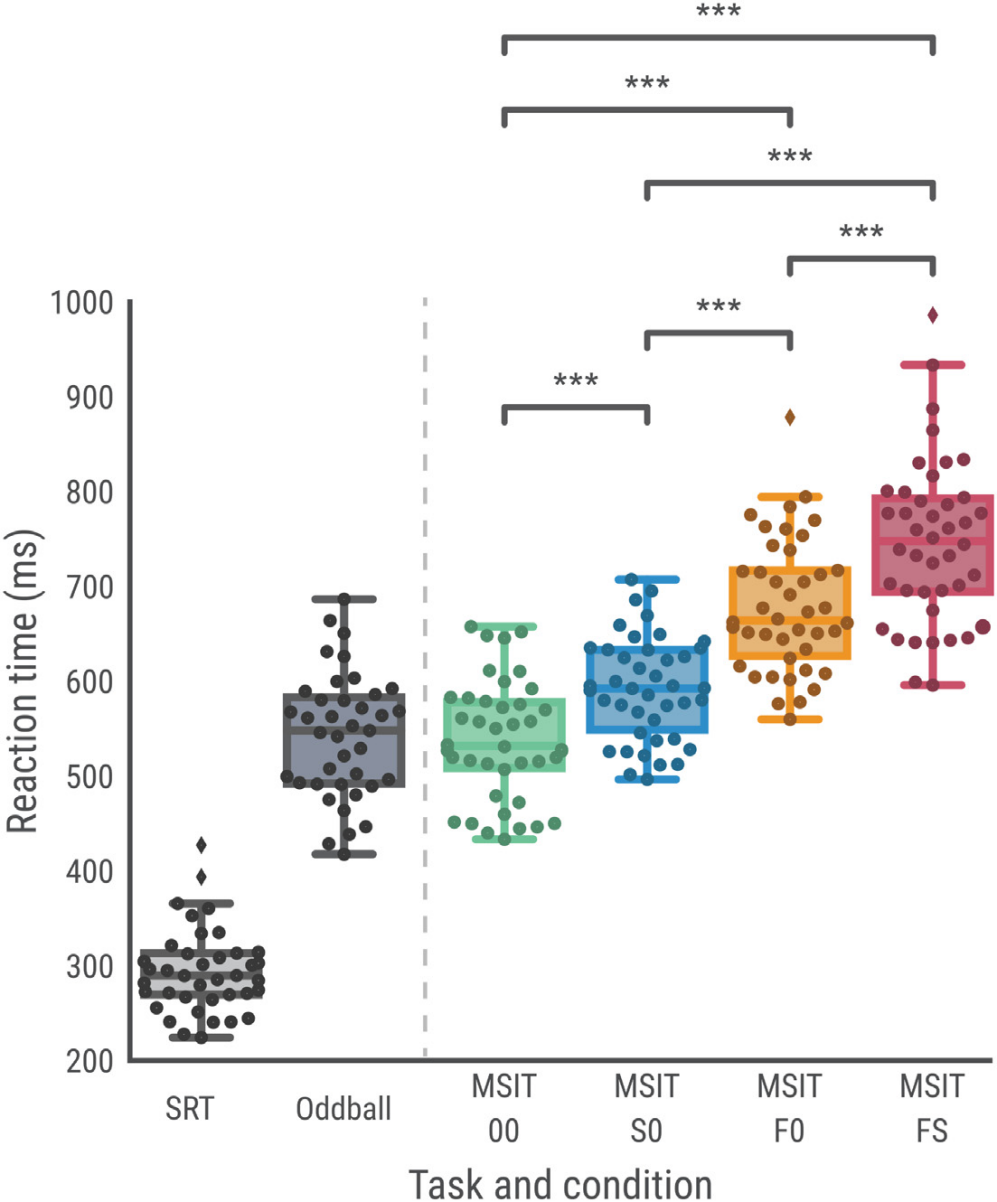
: Reaction time data for SRT, MSIT, and oddball tasks. Symbols: *** < 0.001.

**Table 1:** : Demographic and neuropsychological data for 42 participants

Parameter	Mean ± SD	Mode	Median
**Demographic and health characteristics**			
Age (years)	24.62 ± 4.12		
Medication use at the time of the experiment (%)	11.9		
Caffeine use prior to the experiment (%)	40.48		
**Emotional functioning**			
Subjective stress level (1–5)^1^	2.76 ± 1.08	2	3
Subjective rest level (1–5)^1^	3.45 ± 0.99	4	4
UMACL/UWIST HT (10–40)^2^	31.50 ± 5.77	36	34
UMACL/UWIST EA (9–36)^3^	29.43 ± 5.56	30	30
UMACL/UWIST TA (10–40)^4^	14.72 ± 4.75	11	14

1 5-point Likert-type scale: 1 = “low level of stress” and “poor rest”; 5 = “high level of stress” and “well slept and rested,” respectively.

2 Hedonic tone scale: 10: low, 40: high.

3 Energetic arousal scale: 9: low, 36: high.

4 Tense arousal scale: 10: low, 40: high.

Each participant took psychometric tests directly related to the EEG recording session. Handedness was verified with the Edinburgh Handedness Inventory [20]. Additionally, each participant was required to get sufficient sleep and come to the study visit well rested.

Questions about their subjective rest and stress levels were included in the questionnaire completed before the EEG session along with demographic (age, sex) and basic health information (medication use, type of medication, caffeine uptake, phase of the menstrual cycle). Additionally, mood was measured with the Polish version [21] of the UWIST Mood Adjective Check List (UMACL) [22], which measures hedonic tone (HT) and energetic arousal (EA) subscales, which are related to subjective feeling of pleasantness and energy needed for any activity, and the tense arousal (TA) subscale, which is associated with fear and tension (Table 1).

The Amsterdam Resting-State Questionnaire (ARSQ) [23] was completed immediately following the resting-state recording. The ARSQ 1.0 version, which was applied, is a 50-item self-report questionnaire that tracks participants’ ongoing thoughts and feelings and is used to structure measurements of resting-state cognition. All items were scored on a 5-point Likert scale, where 1 = “completely disagree,” 2 = “disagree,” 3 = “neither agree nor disagree,” 4 = “agree,” and 5 = “completely agree.” The ARSQ 1.0 has demonstrated good validity and reliability [22]. For the purposes of this project, we prepared the Polish version of the questionnaire. All items have been translated and back-translated. The translation was assessed by 3 judges for compliance with the original version (Polish version available on request). Internal consistency (Cronbach α) for the total scale comprising all items (50) reached acceptable value (.760). According to the model comprising 27 items of ARSQ 1.0 [22], Cronbach α were also calculated, separately, for 7 dimensions: Discontinuity of Mind (.773), Theory of Mind (.642), Self (.632), Planning (.773), Sleepiness (.806), Comfort (.849), and Somatic Awareness (.602). Internal consistency for the total scale was .800 for the 27-item model. Note the item “I had my thoughts under control” was reverse coded (for details see [23]).

#### Recording software and devices

EEG data were recorded using an actiCHamp amplifier system (Brain Products GmbH, Munich, Germany) and Brain Vision Recorder. High-density actiCAPs with 128 active electrodes were used (actiCAP, Brain Products, Munich, Germany). A standard 128-channel electrode configuration was used (files are available through the manufacturer website, brainproducts.com). The on-line reference was set to FCz, which can be recalculated to any off-line reference type, including single or averaged earlobes (channel TP9 recorded from left earlobe and TP10 from right earlobe). At the end of each session, real electrode locations were measured with a handheld CapTrak (Brain Products GmbH, Munich, Germany) 3D scanner. Impedance was reduced to ∼5 kΩ (Table 4) by careful gel application (Supervisc, extra viscous gel) and skin rubbing. Sampling rate was set to 1,000 Hz, low-pass filter was set to 280 Hz, and no high-pass or Notch filters were used during recording.

#### Environment

The EEG experiments were conducted in the Nencki Institute of Experimental Biology PAS, in the EEG laboratory, and started either in the morning (9 AM) or early afternoon (1 PM). The participants had the possibility to choose the most convenient time session and to get enough sleep to be well rested for the recording time. Each EEG session took ∼3 hours, which included signing documents, participant preparation, and task execution (Table 2). To reduce testing time, the 128-electrode cap was prepared before the participant arrived in the laboratory. All EEG sessions were conducted in a quiet, comfortable room with a dim light. The participant was seated in a chair with armrests, and the chair was facing the front of a monitor. The researcher supervised the study from an adjacent room via remote desktop connection to the recording computer and a LAN camera overlooking the EEG laboratory.

**Table 2:** : Experimental procedure details

No.	Task	Recorder runs (No.)	Saved files (No.)	Duration (min)
1.	Consent and additional documents (including UMACL/UWIST Mood Adjective Check List and a questionnaire measuring the level of stress and relaxation)			10–15
2.	EEG preparation			60–90
3.	Resting-state instruction and recording	2	1	10
4.	Resting-state questionnaire			∼5
5.	Simple reaction time instruction and recording	2	1	7
6.	MSIT+ instruction and training			∼5
7.	MSIT+ recording	4	1	22
8.	Oddball instruction and training			∼5
9.	Oddball recording	4	1	22
10.	CapTrak session	1	1	15

#### Experiment and datasets

Experiment procedures, including the duration and number of recorded data files, are listed in Table 2.

Participants were given printed instructions before each task and the experimenter answered all related questions. Training for MSIT+ and oddball tasks was performed by each participant before the main task. In both ∼45–50 stimuli were presented. The MSIT+ training lasted for ∼2.5 min, whereas oddball training lasted ∼1.3 min. The participant went through the training until the instructions were completely understood, which usually took one session.

In all tasks, stimuli were presented using Presentation^®^ software (Version 20.2, Neurobehavioral Systems, Inc., Berkeley, CA). Distance from the screen was maintained for all participants: 55 cm (∼21.6 in) so that stimuli size in SRT and MSIT tests was ∼1° of visual angle vertically (as indicated in the MSIT recommendations [15]). Vertical visual angle in oddball was ∼1.07° for standard/distractor stimuli and ∼1.22° for target ones. Stimuli were presented on a dark screen (RGB: 48, 48, 48) with gray font color (RGB: 226, 226, 226).

#### Task details

##### Extended multi-source interference task (MSIT+)

**Figure 2:**
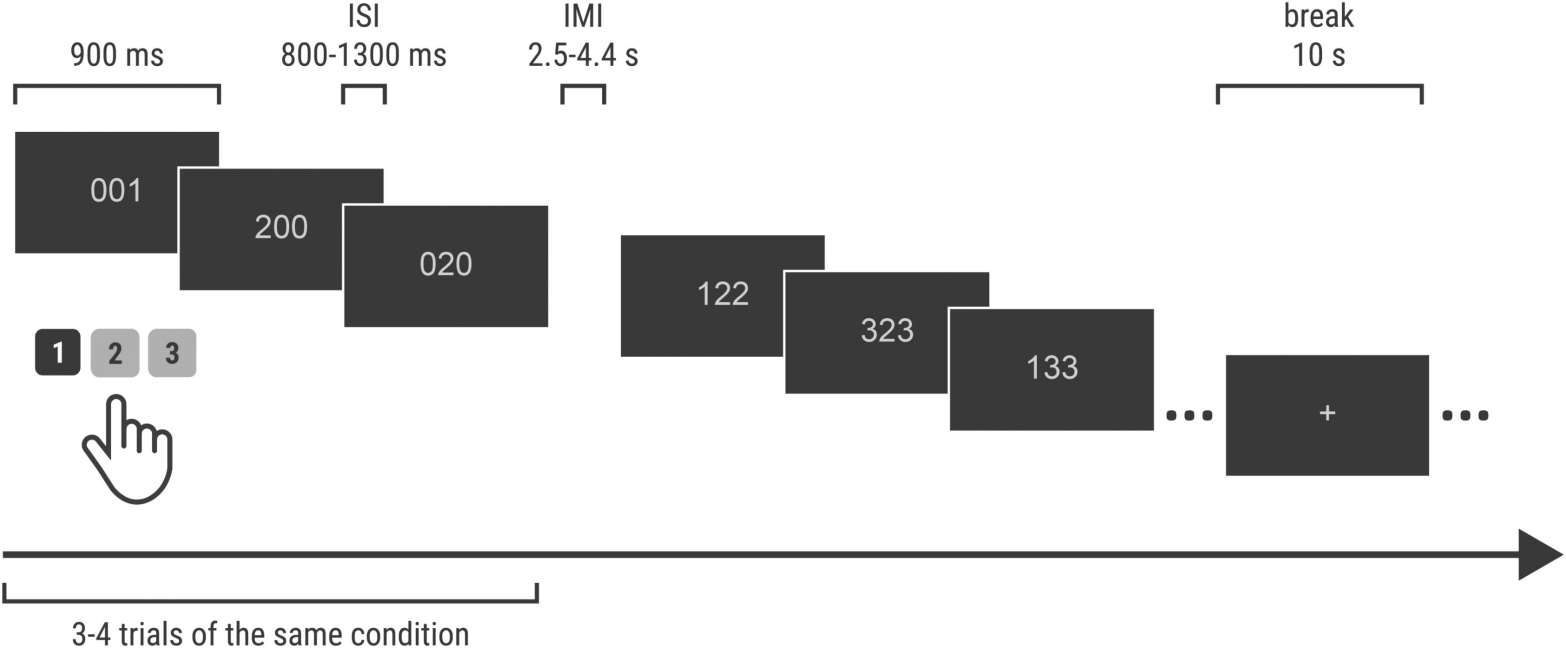
: MSIT+ task design. ISI: interstimulus interval; IMI: inter-mini-block interval.

The extended MSIT contains 4 conditions: 00 (no interference, non-conflict), S0 (Simon effect), F0 (Flanker effect), and FS (double-conflict, Simon and Flanker effects combined) [14, 15, 24]. Three digits are displayed in the center of the screen, including 2 digits that are always identical and 1 deviant. Participants are instructed to indicate the deviant digit in each set. Possible stimuli were as follows: 00 (100, 020, 003), S0 (010, 001, 200, 002, 300, 030), F0 (122, 133, 121, 323, 113, 223), FS (212, 313, 221, 331, 112, 211, 332, 233, 311, 322, 131, 232). Responses were made with the right (dominant) hand on the numeric keyboard: response 1—“1” key pressed with right index finger; response 2—“2” key pressed with right middle finger; response 3—“3” pressed with right ring finger. The task was presented in a mini-block design format. In each trial, the stimulus was presented for 900 ms and followed an interstimulus interval (ISI) that ranged from 800 to 1,300 ms (in steps of 100 ms) (Fig. 2). Stimuli were separated by a blank screen. Three or 4 stimuli of the same condition were presented consecutively with inter-mini-block intervals (IMI) that ranged from 2.5 to 4.4 s (ISI duration included), between another set of 3–4 stimuli of another condition. Between 30–40 stimuli, a 10 s rest with a white fixation cross centered on the screen was presented. A mean of 399 (SD 6) trials were presented to each participant (mean number of trials for each condition: 00: 102 [SD 7], S0: 105 [SD 7], F0: 94 [SD 9], FS: 98 [SD 9]). Task duration with breaks was set to ∼22 min. Marker denotation is presented in Table 3.

**Table 3:** : Markers used in EEG files for 4 types of task

Marker	Task			
	SRT	Oddball	MSIT+	REST
S1	Response	Response	Response “1”	
S2			Response “2”	
S3			Response “3”	
S5	Stimulus	Standard stimulus	F0 stimuli	
S6		Target stimulus	FS stimuli	
S7		Deviant stimulus	00 stimuli	
S8			S0 stimuli	
S10	Break	Break	Break	Break
S11			End of a mini-block	End of a break
S12	Beginning of the run	Beginning of the run	Beginning of the run	Beginning of the run

##### Oddball task

**Figure 3:**
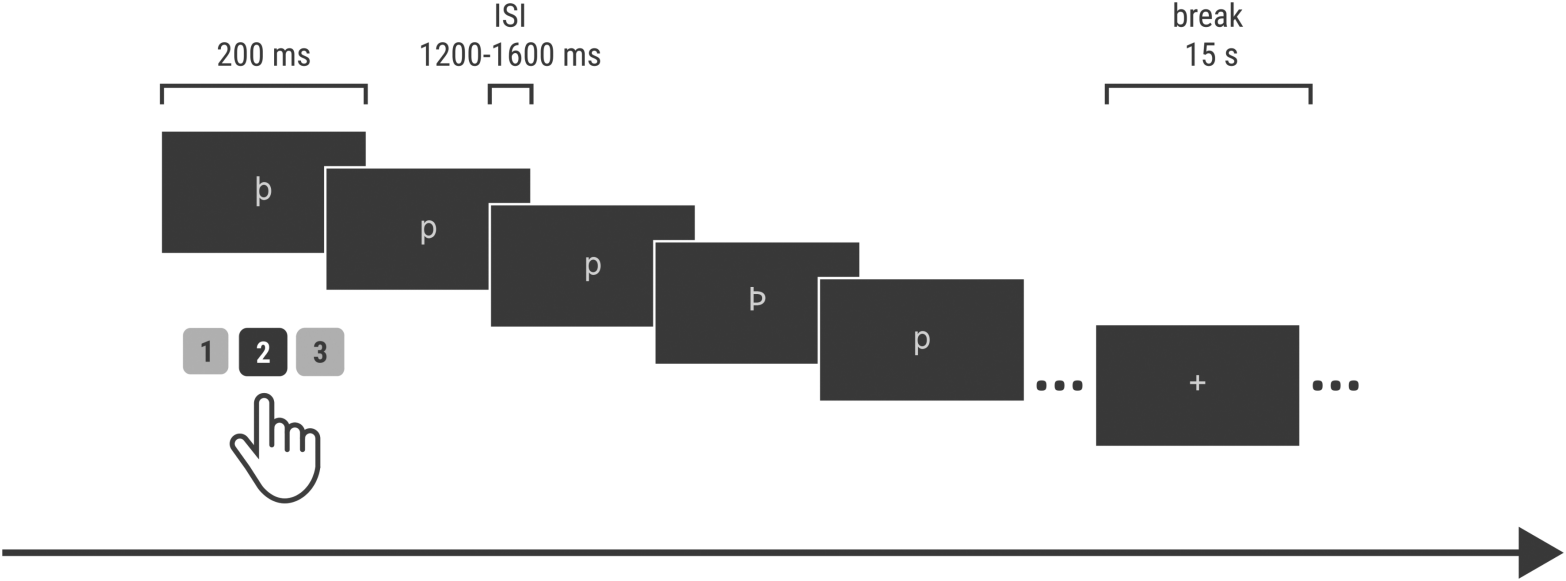
: Odball task design. ISI: interstimulus interval.

The oddball paradigm provides data with well-known ERP components such as P3, which is frequently used in BCI approaches. In this paradigm, 3 stimuli (rare target—þ, rare distractor—Þ, frequent standard—p) were presented in a pseudorandom order with the restriction that 2 rare stimuli could not appear in a row (Fig. 3). Participants were instructed to respond to the target stimulus (press key “2” on numerical keyboard with right middle finger) and inhibit responses to other stimuli. The session contained 660 stimuli, which included ∼12% targets, 12% deviant, and 76% standard stimuli. For 5 participants (i.e., sub_13, sub_23, sub_24, sub_37, sub_38), the number of stimuli in the EEG files (and corresponding *_events.tsv files) is slightly different (from 659 to 775), owing to technical acquisition errors that were solved during the recording. Task duration with breaks was set to ∼22 min. Each stimulus was presented for 200 ms with a 1,200–1,600 ms ISI, and stimuli were separated by a blank screen. Four breaks of 15 s duration were introduced during the task, with a white fixation cross centered on the screen. Marker denotation is presented in Table 3.

##### Simple reaction time task

SRT task data can be easily compared to the results of other tasks and provide a measure of the basic motor and processing speed capabilities of the participants. In particular, the SRT gauges participants’ “average” reaction time in response to visual stimuli, which is considered the most basic measure of processing speed. A simple stimulus (“000”) was displayed (900 ms) centrally on the screen (RGB colors as described above) with 700–1,200 ms ISIs (in steps of 100 ms). The participant was instructed to respond with an index finger (pressing “2” on a numerical keyboard) as fast as possible. On average, 129 trials were presented to each participant. Marker denotation is presented in Table 3.

##### Resting-state session

The REST task is widely used to monitor spontaneous brain activity. In particular, the REST task measures an initial, task-negative state during which no task is given to the participant. REST recording lasted for 10 minutes and was performed in an eyes-open condition. A plus sign was displayed (RGB colors as described above) centrally on a dark screen for gaze fixation. Marker denotation is presented in Table 3.

#### Data format and structure

The data structure was prepared in accordance with BIDS (Brain Imaging Data Structure) rules [25, 26] with the use of EEGLAB [27] to correct and standardize the files. This approach allows for coherent data organization, reduces errors, and makes it possible for different researchers to reuse the datasets for their own purposes and develop automated tools for data analysis. The main folder contains the data for all participants, each in a separate directory named with an arbitrary number (sub-01, sub-02, …, sub-42), and metadata in *.tsv and *.json files. Participants’ “.tsv” and “.json” files describe demographic, psychometric, and health information. Metadata filenames are constructed according to the following rule: “task-[msit/oddball/res/srt]_eeg.json” files describe task along with EEG parameters and “task-[msit/oddball/res/srt]_events.json” files describe event details.

EEG data are provided in Brain Vision Recorder native format (“.eeg,” “.vhdr,” “.vmrk”). Raw behavioral log files are saved in Presentation software's native format (“.log”) and separate event tables based on EEG files are prepared in tab-separated documents (“.tsv”) for each participant/task. The raw, original exact electrode location data are saved in CapTrak format (“.bvct”). Data available in “participants.tsv” file:

- Demographic characteristics: age, sex.
- Health: medication use on the day of experiment, type of medication used, caffeine uptake, phase of the menstrual cycle (for women).
- Subjective stress level, subjective rest level.
- The ARSQ [22] results for each question.
- UMACL/UWIST (Mood Adjective Check List) [21, 22] raw results for each question and recalculated HT, EA, and TA scales.

#### Additional notes

Additional markers of the type “boundary” can be found in all described tasks and files. Events of such type are added automatically by EEGLAB when some portions of the data were deleted, or when portions of continuous datasets are concatenated. Of note, this occurs at the beginning of each run after uploading the data from Brain Products format to EEGLAB. Some raw files were concatenated because of technical errors during recordings, which required saving >1 file for each task. In these files, when a new portion of data was introduced, an additional marker with the code “New Segment” was created. Files that were prepared that way are as follows: sub-17 (MSIT), sub-21 (MSIT), sub-23 (MSIT), sub-25 (REST), sub-37 (oddball), sub38 (oddball, MSIT). Furthermore, 1 participant did not complete the SRT task correctly (sub_29).

## Reliability

### Methods

Behavioral data for the SRT and oddball task were calculated from EEG files (i.e., “*_events.tsv”) and MSIT+ data were calculated from original Presentation (*.log) files. The raw files were used because these provide more precise information about individual stimuli. Trials that were missed (i.e., no responses), anticipation (faster than 100 ms for SRT/oddball task and 200 ms for MSIT task), and longer than the time of stimulus plus the shortest used ITI were coded as errors for all tasks. Additionally, multiple responses to 1 stimulus were also coded as an error in the MSIT+ task. One participant was excluded in the calculation of behavioral data for the SRT task because his/her responses were not recorded correctly (sub_29).

Statistical analysis of reaction times was performed in JASP (version 0.14.1.0) [28]. Repeated-measures ANOVA was conducted with task conditions as repeated-measures factors, and the Shapiro-Wilk test was used to test normal distribution. The assumption of sphericity was violated and Greenhouse-Geisser correction was used.

**Figure 5:**
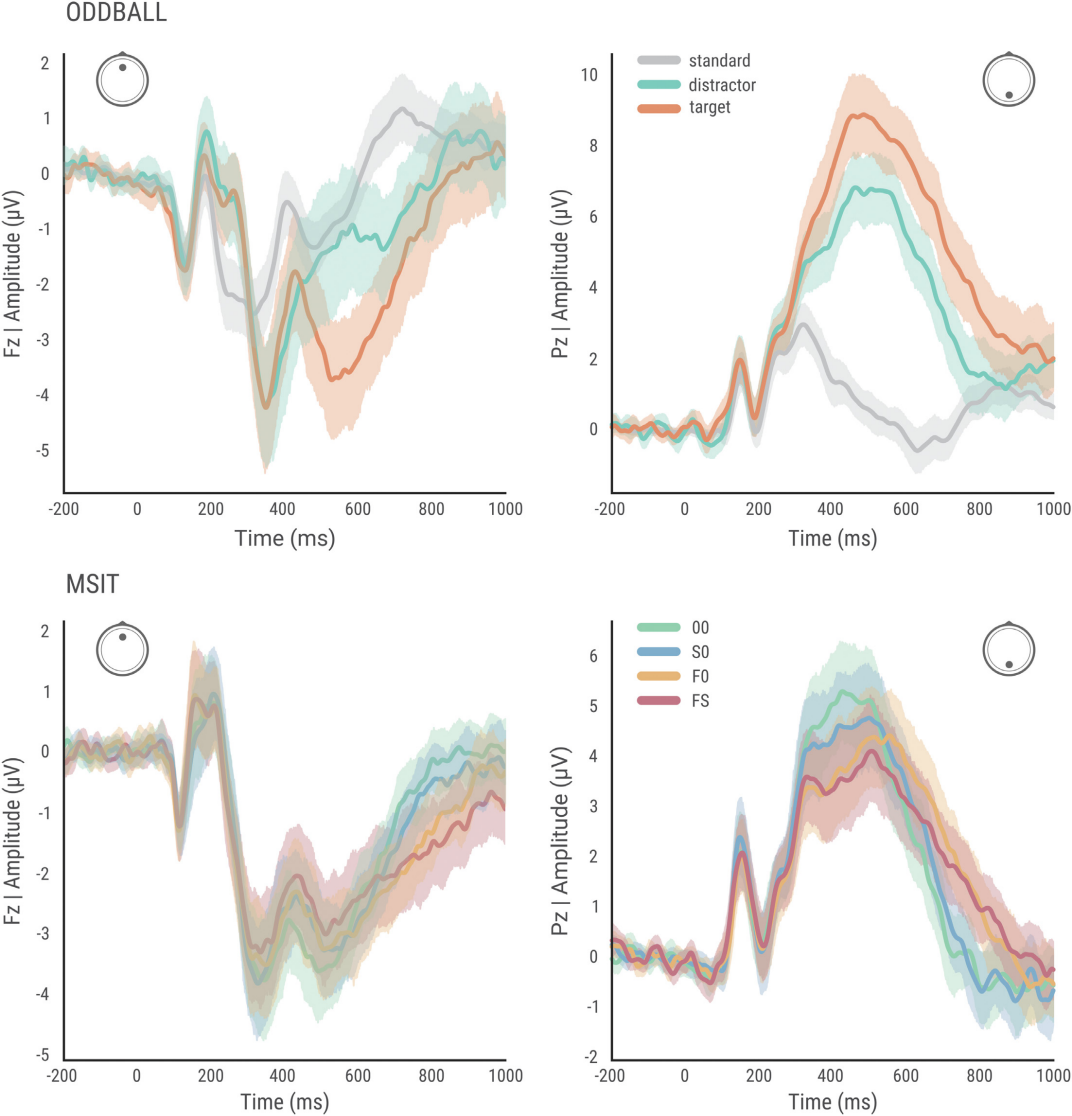
: Grand averaged ERP (with shading indicating SD) for oddball (top) and MSIT+ (bottom) tasks, shown for 2 distinct midline electrodes Fz (more frontal) and Pz (parietal).

Signal artifacts (eye movements, cardiac, muscle artifacts) were corrected for the purpose of ERP plotting (Fig. 5) using Independent Component Analysis (ICA [29]). Remaining artifacts were removed during semi-automatic visual inspection. Data was band-pass filtered (0.5–40 Hz), re-averaged to average reference, and segmented into segments from 200 ms before stimulus presentation to 1,000 ms after stimulus presentation. Clean segments with correct responses were averaged separately for every condition and task. Baseline correction was applied for visualization purposes.

#### Dataset quality

We placed great emphasis on obtaining clean EEG data. Electrode-skin impedance was kept as low as possible (Table 4, mean impedance for MSIT task: 4.66 kΩ [SD 2.36]), and participants were informed about the deteriorating influence of their movements on the EEG quality. We observed recorded EEG signals via remote desktop connection and intervened when necessary. Unavoidably, there are eye-blink artifacts on frontal electrodes, and often muscle artifacts on temporal and occipital poles. A large signal drift is also observed for some participants/electrodes because no high-pass filters were used (this step was omitted to ensure the possibility of further data modifications/filtering according to analytical needs).

**Table 4:** : Mean impedance levels for each task and each participant

Subject ID	Mean impedance level (kΩ)			
	SRT	Oddball	MSIT+	REST
sub-01	4.21	3.64	4.21	4.52
sub-02	3.60	3.57	3.63	3.84
sub-03	4.77	4.71	4.95	5.05
sub-04	3.81	3.84	3.70	3.78
sub-05	6.09	6.09	6.09	6.09
sub-06	4.87	4.73		5.56
sub-07	3.98	3.23	3.98	4.53
sub-08	4.78	4.78	4.78	4.78
sub-09	3.72	3.60	3.93	4.16
sub-10	5.07		5.07	5.50
sub-11	5.79	5.94	6.00	6.20
sub-12	5.20	5.15	5.19	5.16
sub-13			5.43	5.88
sub-14	2.02	2.02	2.64	3.62
sub-15	3.95	4.07	4.20	4.53
sub-16	2.19	2.16	2.50	2.76
sub-17	3.25	3.43	3.42	3.53
sub-18	3.60	3.60	3.93	4.53
sub-19	2.26	2.49	2.18	2.53
sub-20	2.98	2.75	3.48	4.60
sub-21	2.67	2.58	3.09	3.77
sub-22	4.47	4.47	4.47	5.09
sub-23	8.27	7.87	8.71	9.43
sub-24	10.96	10.88	11.49	11.50
sub-25	3.48	2.94	3.48	4.18
sub-26	2.34	2.13	2.57	3.17
sub-27	4.80	4.79	4.61	5.02
sub-28	2.54	2.45	3.03	3.98
sub-29	6.66	6.23	5.52	
sub-30	4.05	3.53	5.04	5.83
sub-31	3.29	3.11	3.80	4.23
sub-32	14.74	14.66	15.01	
sub-33	4.48	4.33	5.12	5.91
sub-34	4.13	4.09	4.89	4.72
sub-35	4.32	3.69	4.24	4.34
sub-36	3.94	3.68	4.13	4.47
sub-37	2.85	2.74	2.92	3.14
sub-38	3.81		3.67	3.97
sub-39	2.96	2.75	2.92	3.22
sub-40	6.27	6.27	6.27	6.27
sub-41	3.57	3.60	3.89	3.95
sub-42	3.16	3.26	2.91	2.96

Data preprocessing for the purpose of our own analyses (not included in the database) indicated relatively good quality of EEG signal. Indeed, on average, in the most restrictive and conservative approach, we excluded 22.1% (SD 14) of trials.

#### Dataset basic results

The basic behavioral and ERP results also confirm the quality of this dataset. Indeed, reaction times and accuracy corresponded with the increasing difficulty of task conditions, as expected. Furthermore, typical ERP waves were obtained, as expected, for attention and cognitive control tasks (e.g., N200, P300, N450).

Responses in SRTs are usually faster than responses in any choice reaction tasks. As expected, our mean SRT latencies were fast (Table 5 and Fig. 4), and were faster than observed in the 00 (no conflict) MSIT condition, and as compared to oddball responses. In addition, participants made few errors: 4.23%, including 2.12% related to response failures and only 1.9% related to premature responses (i.e., faster than 100 ms). The rest of the errors corresponded with late/long responses.

**Table 5:** : Summary of behavioral results

Parameter	Mean ± SD
**SRT**	
Reaction time to stimulus (ms)	294.69 ± 43.28
**Oddball**	
Reaction time to targets (ms)	542.13 ± 65.50
Target omission (%)	5.60 ± 8.33
False alarm (%)	
To distractors	9.52 ± 8.90
To standards	0.24 ± 0.46
**MSIT+**	
Reaction time (ms)	
To 00	537.29 ± 62.11
To S0	592.61 ± 54.48
To F0	678.71 ± 69.80
To FS	746.82 ± 85.87
Response errors (%)	
00	0.90 ± 2.85
S0	0.69 ± 2.22
F0	1.23 ± 3.55
FS	1.55 ± 3.83

Participants for SRT: N = 41; Oddball: N = 39; MSIT: N = 42..

Table 5 summarizes the behavioral data. Three participants most likely misunderstood or forgot the oddball instructions and frequently responded to distractor/deviant stimuli (sub_02–false alarm to distractors = 98.77%; sub_14–false alarm to distractors = 97.53%; sub_17–false alarm to distractors = 76.54%). These 3 participants were excluded from the behavioral summary of the oddball task provided in Table 5. Stimuli similarity in the oddball paradigm made the task quite difficult for participants, as they committed a mean of 9.52% (SD 8.90) of errors towards distractors.

The gradual increase of the conflict over the 4 conditions of the MSIT+ is clearly seen in reaction times (00 < S0 < F0 < FS, Fig. 4; *F*(1.76, 72.02) = 391.15, *P* < 0.001; all post hoc pairwise comparisons significant with *P* < 0.001 and Bonferroni correction). As expected, more response errors were observed for the most demanding conditions, e.g., more errors were observed for F0 (mean 1.23% [SD 3.55]) and FS (mean 1.55% [SD 3.83]) as compared with 00 (0.90% [SD 2.85]) and S0 (0.69% [SD 2.22]) conditions. However, 16 of 42 participants (38%) did not commit any errors, and another 19 committed 1–3 errors during the entire task. Therefore, a total of 83% of participants did not commit >3 errors during the whole task. Only 4 participants committed substantially more errors during the task (>17). Given these observations, more elaborate error-rate analyses are neither possible nor recommended.

#### Dataset ERP results

The aim of this section was to show basic characteristics of the obtained signal. During both the MSIT+ and oddball tasks, the primary ERPs are clearly visible (Fig. 5).

The most studied component in healthy and clinical populations is P3, which peaks between 300 and 600 ms after stimulus onset [30]. P3 closely reflects attentional and memory processes in the human brain [30] and is often studied in the context of an oddball task. In our odball task results, much higher amplitude of P3 was visible for target in comparison to distractor and standard stimuli, especially for electrodes placed in parietal areas (such as Pz), which agrees with the current literature [31]. Additionally, there is a clear distinction between 2 P3 subcomponents—namely, P3a and P3b—in our 3-stimulus oddball task. The P3a is more likely to be evoked during tasks with novelty, whereas P3b is considered to reflect attention [31]. Another well-studied component related to cognitive control, attention, and conflict resolution is the frontocentral negative peak (N2). The N2 is commonly observed between 200 and 350 ms after stimulus onset [32]. A clear frontocentral N2 is observed in our data collected during the oddball task (Fig. 5). In MSIT+ ERPs, a later conflict-related negativity (i.e., the N450) is observed on frontocentral electrodes (Fig. 5). Of note, the N450 extends more posteriorly as a negative deflection within the P3. The P3b and N450 clearly discriminate between the 4 conditions of MSIT task. In particular, the P3 shows higher amplitudes for faster responses (00 > S0 > F0 > FS) whereas the N450 amplitude increases with increasing levels of conflict (00 < S0 < F0 < FS). Yet another conflict-related wave is clearly visible in our MSIT data: the slow potential (SP). The SP is commonly observed after a crossover point on the descending slope of a waveform [33]. The conflict SP amplitude increases with increasing levels of conflict (00 < S0 < F0 < FS), as expected from earlier literature [34]. These basic results show that the tasks were designed correctly.

Additionally, we used MSIT and SRT single-trial EEG data to detect and discriminate between attentive brain states with use of machine learning methods [35]. We have used multisource interference trials (FS) from MSIT as a condition with high attentional load, and SRT trials as a low attentional load. Classification accuracy between high-attention and low-attention conditions was up to 100% for individual participants, with 89% average classification accuracy for all participants [35], which validates the tasks used and proves high quality of the obtained data and their general usefulness in different analytic approaches.

## Summary and Perspectives

It is essential to integrate and reuse data to improve the reliability of results in neuroscience. Our Nencki-Symfonia EEG dataset is well suited for neuropsychological research regarding common and distinct mechanisms involved in different cognitive tasks. A wide range of classical EEG/ERP features can be compared across tasks for any chosen subset of electrodes. At the same time, full high-density EEG recording allows for source localization and detailed connectivity studies. Neurophysiological measures can be also correlated with the psychometric data. This dataset can also be used to develop and verify novel analytical and classification approaches, including advances in the field of deep/machine learning algorithms, recognition of single-trial ERP responses to different task conditions, and detection of particular EEG/ERP features for use in BCI applications. BCI as a discipline could be placed between neuroscience and computer science, and brings new solutions for the disabled or ill, as well as for healthy individuals. Most studies tend to focus on 2 main solutions: (i) BCIs based on movement control for preparation of robotic limbs with need of motor imagery tasks and (ii) attentional-based BCIs used to create direct communication systems, which are often based on steady-state visual evoked potential. However, these paradigms may not always work for all patients, especially those in complete locked-in syndrome (i.e., individuals who do not exhibit eye movements). Furthermore, there is a need to modernize our approach to BCIs and develop gaze-independent approaches. Active eye movements are not necessary when stimuli are presented sequentially. For example, the P300 component is elicited by stimuli that draw participant attention and is frequently used for BCI control [36, 37]. The development of improved BCI requires a deeper understanding of the basics of EEG signals, functioning of brain areas, and the connections between them, with a strong emphasis on attention control. Some approaches have attempted to use conflict tasks (e.g., semantically congruent and incongruent stimuli) to first, evaluate performance, and then, build BCI systems for patients with disorders of consciousness [37]. As seen above, BCIs still have many challenges that could be addressed with a more robust system training that would allow better accuracy. A more robust system would also improve the ability of the system to learn to “read” real brain signals between nonstationary noises and artifacts.

Deep/machine learning approaches are widely used to detect specific patterns of EEG activity and improve understanding of brain functions [38, 39, 40, 41, 42]. These approaches are promising for developing new medical methodologies for early intervention and treatment of various brain dysfunctions, such as depression, stress-induced conditions, Alzheimer disease, autism spectrum disorder, attention deficit hyperactivity, and more [43]. Neural attention models are the most recent state-of-the-art deep learning approaches that show promise in this area [44]. There is also a critical need to improve feature extraction, i.e., the process of analyzing signals to distinguish signal features from extraneous content. The proposed dataset could serve as a benchmark and help to evaluate the performance of several novel classifiers in an off-line scenario. Such a process is frequently used to evaluate new approaches (cf. [45]).

Advances in the ability to decode mental and cognitive states is a vital branch of neuroscience and computer science that has gained new attention in recent years [46, 47, 48, 49]. Approaches based on machine learning can decode task engagement, performance, and attention from MSIT and oddball tasks, which can be vital for BCI and novel systems that could detect early mental fatigue. This could be used as a biomarker of fatigue, especially in professions where there is a need for constant attention (e.g., air traffic controllers, professional drivers). Resting-state brain dynamics have been shown to predict the effects of oddball paradigms. For example, rest regional power of a few brainwaves has been shown to correlate with the latency and amplitude of P300, N3, P2, and N1 components [50].

All the tasks included in this dataset can also be used for the in-depth study of the so-called “time-on-task” (time spent to solve the task) problem [51]. The “time-on-task” concept, in part, appears to oppose the results of conflict processing studies. For example, it has been asserted that the dorsal medial frontal cortex is more sensitive to time-on-task rather than conflict itself.

### Summary of the Advantages of This Dataset

- Localizer data of individual electrode locations, which can greatly improve the reliability of EEG source analysis given that real electrode localization can slightly vary from standard templates and across participants owing to differences in head geometry;
- recorded A1 and A2, which enables the off-line possibility of using classical ear-referenced montages;
- high-density recording, which allows for in-depth connectivity and source localization studies;
- 3 different tasks (and additional resting-state protocol) completed by the same participants;
- the first publicly available dataset that includes the MSIT in healthy participants;
- corresponding resting-state questionnaire along with EEG resting-state data;
- relatively large sample size (N = 42);
- 2-fold verification of the data via behavioral and simple ERP investigation;
- detailed health data about participants (menstrual cycle phase, medication, and caffeine use).

### Summary of the Limitations of This Dataset

- Relatively short durations of stimuli and ISIs may not be optimal to study induced oscillatory effects, which may be of interest to some researchers, as shown previously [52, 53].
- Lack of an open access to fMRI dataset from the same task and participants is a limitation, compromising the cross-methodological comparison of MSIT+ results. However, such dataset will be made public with the upcoming fMRI results article from our research partners.

## Data Availability

All the data underlying this article, including EEG datasets, behavioral, and basic questionnaire data, are available in the Nencki-Symfonia EEG/ERP *GigaScience* repository, GigaDB [54].

## Abbreviations

ANOVA: analysis of variance; ARSQ: Amsterdam resting-state questionnaire; BCI: brain-computer interface; EEG: electroencephalography; ERP: event-related potential; fMRI: functional magnetic resonance imaging; ISI: interstimulus interval; LAN: local area network; MSIT+: extended multi-source interference task; IMI: inter mini-block interval; REST: resting-state; SP: slow potential; SRT: simple reaction task; UWIST: Mood Adjective Check List (UMACL).

## Ethics, Consent, and Permissions

This study was performed in accordance with the declaration of Helsinki and was approved by the Research Ethics Committee, Faculty of Humanities, Nicolaus Copernicus University in Toruń, Poland (No. 6/2018). All participants provided written informed consent to participate in the study and received cash remuneration (200 PLN, ∼44 EUR). Participants signed the consent that granted safety of personal data, with the information that experimental data themselves will be anonymized for use in analyses and publications related to the research project.

## Funding

This work was funded by the Polish National Science Centre (NCN) grant No. 2016/20/W/NZ4/00354 (consortium principal investigator: prof. Andrzej Cichocki). The funding body has not participated at any stage in study design, data collection, analysis, or interpretation.

## Competing Interests

The authors declare that they do not have any competing interests.

## Authors’ Contributions

All authors designed the MSIT and SRT experiments, J.D. and J.W. designed the oddball experiment, J.D. prepared the Polish version of the ARSQ, J.W. implemented the experiments, P.D. and I.A. acquired the data, P.D. and I.A. performed data analysis, P.D. wrote the first draft of the manuscript and I.A., J.D., and E.K. reviewed the manuscript, all authors read and approved the final manuscript, and E.K. supervised the entire study.

## Supplementary Material

giac015_GIGA-D-21-00298_Original_Submission

giac015_GIGA-D-21-00298_Revision_1

giac015_Response_to_Reviewer_Comments_Revision_1

giac015_Reviewer_1_Report_Original_Submission Alex Wiesman -- 11/16/2021 Reviewed

giac015_Reviewer_1_Report_Revision_1 Alex Wiesman -- 1/9/2022 Reviewed

giac015_Reviewer_2_Report_Original_Submission Georgios Dimitrakopoulos -- 11/18/2021 Reviewed

giac015_Reviewer_2_Report_Revision_1 Georgios Dimitrakopoulos -- 1/10/2022 Reviewed
