# Supplementary material for: The Nencki-Symfonia electroencephalography/event-related potential dataset: Multiple cognitive tasks and resting-state data collected in a sample of healthy adults
Source: Gigascience. 2022 Mar 7;11:giac015. doi: 10.1093/gigascience/giac015 (PMC8900497; doi:10.1093/gigascience/giac015)

GigaScience

The Nencki-Symfonia EEG/ERP dataset: Multiple cognitive tasks and resting-state data collected in a sample of healthy adults

--Manuscript Draft--

**Manuscript Number:**	GIGA-D-21-00298
**Full Title:**	The Nencki-Symfonia EEG/ERP dataset: Multiple cognitive tasks and resting-state data collected in a sample of healthy adults
**Article Type:**	Data Note
**Funding Information:**	Narodowe Centrum Nauki (2016/20/W/NZ4/00354) — Not applicable
**Abstract:**	Abstract<br><br>Background<br>One of the primary goals of neuropsychology is to understand the brain mechanisms underlying various aspects of attention and cognitive control. This body of research has developed multiple tasks which have been described in several studies and indicate patterns of both similar and divergent results. A reliable comparison of the data and a synthesis of study conclusions has been precluded by multiple methodological differences. Here, we describe a publicly available, high-density electroencephalography (EEG) dataset obtained at the Nencki Institute of Experimental Biology from a sample of 42 healthy young adults while they performed four cognitive tasks: (1) an extended Multi-Source Interference Task (MSIT+) with control, Simon, Flanker, and multi-source interference trials; (2) a 3-stimuli oddball task with frequent standard, rare target, and rare distractor stimuli; (3) a control, simple reaction task (SRT); and (4) a resting-state task (REST). Basic demographic and psychometric information are included within the dataset (including The Amsterdam Resting-state Questionnaire (ARSQ) and UWIST Mood Adjective Check List questionnaires).<br>Dataset validation<br>First, data validation confirmed acceptable quality of the obtained EEG signals. Typical event-related potential (ERP) waveforms were obtained, as expected for attention and cognitive control tasks (i.e., N200, P300, N450). Behavioral results showed the expected progression of reaction times and error rates, which confirmed the effectiveness of the applied paradigms.<br>Conclusions<br>This dataset is well suited for neuropsychological research regarding common and distinct mechanisms involved in different cognitive tasks. Using this dataset, researchers can compare a wide range of classical EEG/ERP features across tasks for any selected sub-set of electrodes. At the same time, 128-channel EEG recording allows for source localization and detailed connectivity studies. Neurophysiological measures can be correlated with additional psychometric data obtained from the same participants. This dataset can also be used to develop and verify novel analytical and classification approaches that can advance the field of deep/machine learning algorithms, recognition of single-trial ERP responses to different task conditions, and detection of EEG/ERP features for use in brain-computer interface (BCI) applications.
**Corresponding Author:**	Patrycja Dzianok, MSc<br>Nencki Institute of Experimental Biology PAS: Instytut Biologii Doswiadczalnej im Marcelego Nenckiego Polskiej Akademii Nauk<br>Warsaw, POLAND
**Corresponding Author Secondary Information:**	
**Corresponding Author's Institution:**	Nencki Institute of Experimental Biology PAS: Instytut Biologii Doswiadczalnej im Marcelego Nenckiego Polskiej Akademii Nauk
**Corresponding Author's Secondary Institution:**	
**First Author:**	Patrycja Dzianok, MSc

Powered by Editorial Manager® and ProduXion Manager® from Aries Systems Corporation

First Author Secondary Information:	
Order of Authors:	Patrycja Dzianok, MSc
	Ingrida Antonova, PhD
	Jakub Wojciechowski, MSc
	Joanna Dreszer, PhD
	Ewa Kublik, PhD
Order of Authors Secondary Information:	
Additional Information:	
Question	Response
Are you submitting this manuscript to a special series or article collection?	No
**Experimental design and statistics**<br><br>Full details of the experimental design and statistical methods used should be given in the Methods section, as detailed in our Minimum Standards Reporting Checklist. Information essential to interpreting the data presented should be made available in the figure legends.<br><br>Have you included all the information requested in your manuscript?	Yes
**Resources**<br><br>A description of all resources used, including antibodies, cell lines, animals and software tools, with enough information to allow them to be uniquely identified, should be included in the Methods section. Authors are strongly encouraged to cite Research Resource Identifiers (RRIDs) for antibodies, model organisms and tools, where possible.<br><br>Have you included the information requested as detailed in our Minimum Standards Reporting Checklist?	Yes
**Availability of data and materials**<br><br>All datasets and code on which the	No

conclusions of the paper rely must be either included in your submission or deposited in publicly available repositories (where available and ethically appropriate), referencing such data using a unique identifier in the references and in the “Availability of Data and Materials” section of your manuscript.<br><br>Have you have met the above requirement as detailed in our Minimum Standards Reporting Checklist?	
If not, please give reasons for any omissions below.<br><br>as follow-up to "**Availability of data and materials**<br><br>All datasets and code on which the conclusions of the paper rely must be either included in your submission or deposited in publicly available repositories (where available and ethically appropriate), referencing such data using a unique identifier in the references and in the “Availability of Data and Materials” section of your manuscript.<br><br>Have you have met the above requirement as detailed in our Minimum Standards Reporting Checklist?<br><br>"	The data are not yet available, as we follow workflow diagram available on the GigaDB website on 'Submisssion Guildelines' page (http://gigadb.org/site/guide). We already created a GigaDB account, therefore we would like to kindly ask you to provide us with the private FTP login to upload our data.

Manuscript

Click here to access/download;Manuscript;The_Nencki-Symfonia_EEGERP_dataset_art_v2.docx

The Nencki-Symfonia EEG/ERP dataset: Multiple cognitive tasks and resting-state data collected in a sample of healthy adults

Dzianok Patrycja[1] [p.dzianok@nencki.edu.pl; corresponding author], Antonova Ingrida[2] [a.antonova@nencki.edu.pl], Wojciechowski Jakub[1][3] [j.wojciechowski@ifps.org.pl], Dreszer Joanna[4] [jdreszer@umk.pl], Kublik Ewa[1] [e.kublik@nencki.edu.pl]

[1] Laboratory of Emotions Neurobiology, Nencki Institute of Experimental Biology PAS, Warsaw, Poland

[2] Laboratory of Neuroinformatics, Nencki Institute of Experimental Biology PAS, Warsaw, Poland

[3] Bioimaging Research Center, Institute of Physiology and Pathology of Hearing, Warsaw, Poland

[4] Institute of Psychology, Faculty of Philosophy and Social Sciences, Nicolaus Copernicus University in Toruń, Toruń, Poland

Abstract

Background

One of the primary goals of neuropsychology is to understand the brain mechanisms underlying various aspects of attention and cognitive control. This body of research has developed multiple tasks which have been described in several studies and indicate patterns of both similar and divergent results. A reliable comparison of the data and a synthesis of study conclusions has been precluded by multiple methodological differences. Here, we describe a publicly available, high-density electroencephalography (EEG) dataset obtained at the Nencki Institute of Experimental Biology from a sample of 42 healthy young adults while they performed four cognitive tasks: (1) an extended Multi-Source Interference Task (MSIT+) with control, Simon, Flanker, and multi-source interference trials; (2) a 3-stimuli oddball task with frequent standard, rare target, and rare distractor stimuli; (3) a control, simple reaction task (SRT); and (4) a resting-state task (REST). Basic demographic and psychometric information are included within the dataset (including The Amsterdam Resting-state Questionnaire (ARSQ) and UWIST Mood Adjective Check List questionnaires).

Dataset validation

First, data validation confirmed acceptable quality of the obtained EEG signals. Typical event-related potential (ERP) waveforms were obtained, as expected for attention and cognitive control tasks (i.e., N200, P300, N450). Behavioral results showed the expected progression of reaction times and error rates, which confirmed the effectiveness of the applied paradigms.

Conclusions

This dataset is well suited for neuropsychological research regarding common and distinct mechanisms involved in different cognitive tasks. Using this dataset, researchers can compare a wide range of classical EEG/ERP features across tasks for any selected sub-set of electrodes. At the same time, 128-channel EEG recording allows for source localization and detailed connectivity studies. Neurophysiological measures can be correlated with additional psychometric data obtained from the same participants. This dataset can also be used to develop and verify novel analytical and classification approaches that can advance the field of deep/machine learning algorithms, recognition of single-trial ERP responses to different task conditions, and detection of EEG/ERP features for use in brain-computer interface (BCI) applications.

Data Description

Background and purpose

Here, we describe a dataset of EEG/ERP signals and metadata which may be useful in at least three domains: a) in-depth neuroscience investigations related to attention and cognitive control, b) testing machine and deep learning algorithms suited for neuroimage data, with a particular emphasis on attention and cognitive control aspects, and c) new approaches for brain-computer interface (BCI) development.

There is a tremendous need for neuroscience datasets to be available to the wider scientific community, which may help to overcome the reproducibility crisis and improve the quality of research (e.g., larger sample sizes, more reproducible findings). Open access to data may also increase the speed of research and reduce the need to collect multiple redundant datasets, such as in computational neuroscience [1, 2, 3, 4].

Research concerning conflict processing and attentional cognitive control are well-established in the neuroscience literature. Such research has focused primarily on characterizing conflict processing and attentional cognitive control using behavioral [5, 6, 7] and neuroimaging [8, 9, 10] approaches. This work has demonstrated two primary sources of cognitive conflict: (1) stimulus-stimulus interference, which arises from flanking stimuli that are similar to each other, but differ from the target stimulus; (2) stimulus-response interference, which arises from spatial (in)compatibility between the target stimulus and the response button positions. The most common tasks used to study these conflict types include the Flanker Task, which evokes the "Flanker effect" [11], and the Simon Task, which evokes the "Simon effect" [12]. Both the Flanker and Simon effects are included in the multi-source interference task (MSIT) [13, 14], which was designed to maximize conflict effects and strongly engage conflict-specific brain areas, including the dorsal anterior cingulate cortex (dACC). The popularity of the MSIT task has risen rapidly over the past 17 years (Fig. 1).

[Figure 1.]

Fig. 1. Number of publications per year that use the multi-source interference task (MSIT), over the past 17 years. This summary includes both original and theoretical works with comments addressing MSIT published in Google Scholar using the keyword "multi-source interference task". Of note, patents are excluded, and citations are included. In total, 868 papers were identified on MSIT as of 16.08.2021.

There are still open questions related to conflict processing studies, including (1) the exact definition of conflict, (2) how conflict influences reaction times, and (3) how conflict is resolved by brain mechanisms. Further, it is unclear if the global phenomenon of 'conflict' actually exists. If yes, it is also unclear whether conflict is resolved by a single, dedicated brain mechanism, regardless of the specific details of the conflicting events. In contrast, conflict may be detected and resolved by different neural mechanisms, depending on the type of conflicting stimuli. A reliable comparison of the data and a synthesis of study conclusions is precluded by

multiple methodological differences. To our knowledge, only two MSIT-based datasets are publicly available: (1) a dataset that includes intracranial EEG with and without brain stimulation collected from 21 patients with epilepsy [15, 16]; (2) a dataset of EEG/deep brain stimulation with affective MSIT collected from 14 patients with obsessive-compulsive disorder (OCD) and 14 patients with major depressive disorder (MDD) [17, 18]. However, these datasets are relatively limited in sample size ($N < 25$). To our knowledge, there are no publicly available MSIT datasets collected in healthy individuals. This precludes the ability to resolve and clarify ongoing and past studies.

Here, we describe an MSIT+ dataset collected in healthy individuals. This database is well suited to address the aforementioned questions because it includes an extended MSIT (MSIT+) task. In addition to non-conflict and multi-source conflict conditions, our task includes two single-conflict conditions (i.e., Simon and Flanker). The same participants performed the classical 3-stimuli visual oddball task with two rare stimuli: a target and a distractor. A simple reaction time task (SRT) was also included as a no-conflict, no-attention, control situation. Prior to completing tasks, EEG data were recorded during a 10 min resting-state (REST) condition. Resting-state paradigms are commonly used in neuroimaging and neurophysiology research to examine spontaneous functional activity of the brain at an individual level.

The included tasks initiate multiple cognitive processes, including attention, attentional control, working memory, and action selection. These tasks are of interest from the perspective of basic science and are also helpful for understanding how their dysfunctions induce various mental and medical conditions. The selected tasks are well suited to answer many co-related questions regarding the brain processes underlying conflict and attentional control. Indeed, we selected two attentional tasks (i.e., MSIT+ and oddball) and the SRT task provides information regarding processing speed of each participant regardless of attentional resources and effort/conflict. The additional REST task is well suited to evaluate task-independent (i.e., spontaneous) activity of

the brain, while psychometric questionnaires (both basic, included in the dataset as well as extended, available on request) allow correlating EEG/ERP results with participant’s individual characteristics. An in-depth analysis of such database (i.e., same participants, same experimental conditions, $N = 42$) may also lead to new clinical applications.

Experimental design

Participants and psychometric measures

Forty-two healthy, right-handed young adults (primarily students; 20-34 years of age, 22 females) completed the described experiment (Tab. 1). Participants were provided with detailed information about the study and a list of exclusionary criteria, including contraindications for EEG/ERP: pregnancy, chronic diseases (e.g., epilepsy, chronic headaches, chronic sleep disorders), skin diseases and allergies (especially head-related), diagnosed mental and neurodevelopmental disorders, head injury, medication use (particularly those that may influence nervous system functioning), alcohol, and psychoactive substance abuse.

Each participant filled psychometric tests directly related to EEG recording session. Handedness was verified with the Edinburgh Handedness Inventory (EHI) [19]. Additionally, each participant was required to get sufficient sleep and come to the study visit well-rested.

Questions about their subjective rest and stress levels were included in the questionnaire filled before the EEG session along with demographic (age, sex) and basic health information (medication use, type of medication, caffeine uptake, phase of the menstrual cycle). Additionally, mood was measured with the Polish version [20] of the UWIST Mood Adjective Check List (UMACL) [21], which measures hedonic tone (HT) and energetic arousal (EA) subscales, which are related to subjective feeling of pleasantness and energy needed for any activity and the tense arousal (TA) subscale, which is associated with fear and tension (Tab. 1).

Table 1. Demographic and neuropsychological data

	Participants (*N*=42)			
	Mean		SD	
**Demographics**				
Age (years)	24.62		4.12	
	Percentage of participants (%)			
**Basic health information**				
Medication use at the time of the experiment	11.9%			
Caffeine use prior to the experiment	40.48%			
	Mean	SD	Mode	Median
**Emotional functioning**				
Subjective stress level (1-5)[1]	2.76	1.08	2	3
Subjective rest level (1-5)[1]	3.45	0.99	4	4
UMACL/UWIST HT (10-40)[2]	31.50	5.77	36	34
UMACL/UWIST EA (9-36)[3]	29.43	5.56	30	30
UMACL/UWIST TA (10-40)[4]	14.72	4.75	11	14

[1] *5-point Likert-style scale: 1 = 'low level of stress' and 'poor rest'; 5 = 'high level of stress' and 'well slept and rested', respectively*
[2] *Hedonic tone scale: 10: low, 40: high*
[3] *Energetic arousal scale: 9: low, 36: high*
[4] *Tense arousal scale: 10: low, 40: high*

The Amsterdam Resting-state Questionnaire (ARSQ) [22] was completed immediately following the resting-state recording. The ARSQ 1.0 version, which was applied, is a 50-item self-report questionnaire that tracks participants' ongoing thoughts and feelings, and is used to structure measurements of resting-state cognition. All items were scored on a five-point Likert-type scale (1–5), where 1 = 'Completely Disagree', 2 = 'Disagree', 3 = 'Neither Agree nor Disagree', 4 = 'Agree', and 5= 'Completely Agree'. The ARSQ 1.0 has demonstrated good validity and reliability [22]. For the purposes of this project, we have prepared the Polish version of the questionnaire. All items have been translated and back-translated. The translation was assessed by three judges for compliance with the original version (the Polish version available on request). Internal consistency (Cronbach's alpha) for the total scale comprising all items (50) reached acceptable value (.760). According to the model comprising 27 items of

ARSQ 1.0 [22], Cronbach's alphas were also calculated, separately, for 7 dimensions: Discontinuity of Mind (.773), Theory of Mind (.642), Self (.632), Planning (.773), Sleepiness (.806), Comfort (.849), Somatic Awareness (.602). Internal consistency for the total scale was .800, for the 27-item model. Note the item "I had my thoughts under control" was reverse coded (for details see [22]).

Recording software and devices

EEG data were recorded using an actiCHamp amplifier system (Brain Products GmbH, Munich, Germany) and Brain Vision Recorder. High-density actiCAPs with 128 active electrodes were used (actiCAP, Brain Products, Munich, Germany). A standard 128-channel electrode configuration was used (files are available through the manufacturer website: brainproducts.com). The on-line reference was set to FCz, which can be re-calculated to any off-line reference type, including single or averaged earlobes (channel TP9 recorded from left earlobe and TP10 from right earlobe). At the end of each session, real electrode locations were measured with a handheld CapTrak (Brain Products GmbH, Munich, Germany) 3D scanner. Impedance was reduced to around 5 kΩ (Tab. 7) by careful gel application (Supervisc, extra viscous gel) and skin rubbing. Sampling rate was set to 1000 Hz, low-pass filter was set to 280 Hz and no high-pass nor Notch filters were used during recording.

Environment

The EEG experiments were conducted in the Nencki Institute of Experimental Biology PAS, in the EEG laboratory, and started either in the morning (9 AM) or afternoon (1 PM). Each EEG session took about ~3 hours, which included signing documents, participant preparation, and task execution (Tab. 2). In order to reduce testing time, the 128-electrode cap was prepared

before the participant arrived in the lab. All EEG sessions were conducted in a quiet, comfortable room with a dim light. The participant was seated in a chair with armrests, and the chair was facing the front of a monitor. The researcher supervised the study from an adjacent room via remote desktop connection to the recording computer and a LAN camera overlooking the EEG laboratory.

Experiment and datasets

Experiment procedures, including the duration and number of recorded data files, are listed in Tab. 2.

Table 2. Experimental procedure details

No.	Task	Runs [no.]	Saved files [no.]	Duration [min]
1.	Consent and additional documents (including UMACL/UWIST Mood Adjective Check List and a questionnaire measuring the level of stress and relaxation)	—	—	10-15
2.	EEG preparation	—	—	60-90
3.	Resting-state instruction and recording	2	1	10
4.	Resting-state questionnaire	—	—	~5
5.	Simple reaction time instruction and recording	2	1	7
6.	MSIT+ instruction and training	—	—	~5
7.	MSIT+ recording	4	1	22
8.	Oddball instruction and training	—	—	~5
9.	Oddball recording	4	1	22
10.	CapTrak session	1	1	15

Participants were given printed instructions before each task and the experimenter answered all related questions. Training versions of MSIT+ and oddball tasks were performed by each participant before the main task.

In all tasks, stimuli were presented using Presentation® software (Version 20.2, Neurobehavioral Systems, Inc., Berkeley, CA). Distance from the screen was maintained for all participants: 55 cm (~21.6 in) so that stimuli size in SRT and MSIT tests was around ~1° of visual angle vertically (as indicated in the MSIT recommendations [14]). Vertical visual angle in oddball was approximately ~1.07° for standard/distractor stimuli and ~1.22° for target ones. Stimuli were presented on a dark screen (RGB: 48, 48, 48) with gray font color (RGB: 226, 226, 226).

Tasks details

Extended multi-source interference task (MSIT+)

The extended MSIT contains four conditions: 00 (no interference, non-conflict), S0 (Simon effect), F0 (Flanker effect), and FS (double-conflict, Simon and Flanker effects combined) [13, 14, 23]. Three digits are displayed in the center of the screen, including two digits that are always identical and one deviant. Participants are instructed to indicate the deviant digit in each set. Possible stimuli were as follows: 00 (100, 020, 003), S0 (010, 001, 200, 002, 300, 030), F0 (122, 133, 121, 323, 113, 223), FS (212, 313, 221, 331, 112, 211, 332, 233, 311, 322, 131, 232). Responses were made with the right (dominant) hand on the numeric keyboard: response 1 — '1' key pressed with right index finger; response 2 — '2' key pressed with right middle finger; response 3 — '3' pressed with right ring finger. The task was presented in a mini-block design format. In each trial, the stimulus was presented for 900 ms and followed an interstimulus interval (ISI) that ranged from 800-1300 ms (in steps of 100 ms) (Fig. 2). Three or four stimuli

of same condition were presented consecutively with inter-mini-block intervals (IMI) that ranged from 2.5 to 4.4 s (ISI duration included), between another set of 3-4 stimuli of another condition. Between 30-40 stimuli, a 10 s rest with a white fixation cross centered on the screen was presented. Task duration with breaks was set to ~22 min. Marker denotation is presented in Tab. 3.

[Figure 2.]

Fig. 2. MSIT+ task design (ISI – interstimulus interval, IMI – inter-mini-block intervals)

Table 3. Markers used in EEG files

**Marker**	**Task**			
	**MSIT**	**Oddball**	**SRT**	**REST**
S1	Response ‘1’	Response	Response	—
S2	Response ‘2’	—	—	—
S3	Response ‘3’	—	—	—
S5	F0 stimuli	Standard stimulus	Stimulus	—
S6	FS stimuli	Target stimulus	—	—
S7	00 stimuli	Deviant stimulus	—	—
S8	S0 stimuli	—	—	—
S10	Break	Break	Break	Break
S11	End of a mini-block	—	—	End of a break
S12	Beginning of the run	Beginning of the run	Beginning of the run	Beginning of the run

Oddball task

The oddball paradigm provides data with well-known event related potential (ERP) components such as P3, which is frequently used in BCI approaches. In this paradigm, three stimuli (rare target — þ, rare distractor — Þ, frequent standard — p) were presented in a pseudorandom

order with the restriction that two rare stimuli could not appear in a row (Fig. 3). Participants were instructed to respond to the target stimulus (press key ‘2’ on numerical keyboard with right middle finger) and inhibit responses to other stimuli. The session contained 660 stimuli, which included approximately 12% targets, 12% deviant, and 76% standard stimuli. For five participants (i.e., sub_13, sub_23, sub_24, sub_37, sub_38), the number of stimuli in the EEG files (and corresponding *_events.tsv files) is slightly different (from 659 to 775), due to technical acquisition errors that were solved during the recording. Task duration with breaks was set to ~22 min. Each stimulus was presented for 200 ms with an 1200-1600 ms ISI. Four breaks of 15 s duration were introduced during the task, with a white fixation cross centered on the screen. Marker denotation is presented in Tab. 3.

[Figure 3.]

Fig. 3. Odball task design (ISI – interstimulus interval)

Simple reaction time task

SRT (simple reaction time) task data can be easily compared to the results of other tasks and provides a measure of the basic capabilities of the participants. In particular, the SRT gauges participants’ ‘average’ reaction time in response to visual stimuli, which is considered the most basic measure of processing speed. A simple stimulus (‘000’) was displayed (900 ms) centrally on the screen (RGB colors as described above) with 700-1200 ms ISIs (in steps of 100 ms). The participant was instructed to respond with an index finger (pressing ‘2’ on a numerical keyboard) as fast as possible. On average, 129 trials were presented to each subject. Marker denotation is presented in Tab. 3.

Resting-state session

The REST task is widely used to monitor spontaneous brain activity. In particular, the REST task measures an initial, task-negative state during which no task is given to the subject. REST recording lasted for 10 minutes and was performed in an eyes-open condition. A plus sign ‘+’ was displayed (RGB colors as described above) centrally on a dark screen for gaze fixation. Marker denotation is presented in Tab. 3.

Data format and structure

The data structure was prepared in accordance with BIDS (Brain Imaging Data Structure) rules [24, 25] with the use of EEGLAB [26] to correct and standardize the files. This approach allows for coherent data organization, reduces errors, and makes it possible for different researchers to re-use the datasets for their own purposes and develop automated tools for data analysis. The main folder contains the data for all participants, each in a separate directory named with an arbitrary number (sub-01, sub-02, …, sub-42), and metadata in *.tsv and *.json files. Participants’ ‘.tsv’ and ‘.json’ files describe demographic, psychometric, and health information. Metadata filenames are constructed according to the following rule: ‘task-[msit/oddball/res/srt]_eeg.json’ files describe task along with EEG parameters and ‘task-[msit/oddball/res/srt]_events.json’ files describe events details.

EEG data are provided in Brain Vision Recorder native format (‘.eeg’,. ‘vhdr’,. ‘vmrk’). Raw behavioral log files are saved in Presentation software’s native format (‘.log’) and separate event tables based on EEG files are prepared in tab-separated documents (‘.tsv’) for each participant/task. The raw, original exact electrode location data is saved in CapTrak format (‘.bvct’).

Data available in 'participants.tsv' file:

- Demographic: age, sex.
- Health: medication usage on the day of experiment, type of medication used, caffeine uptake, phase of the menstrual cycle (for women).
- Subjective stress level, subjective rest level.
- The ARSQ [22] results for each question.
- UMACL/UWIST (Mood Adjective Check List) [20, 21] raw results for each question and re-calculated HT, EA and TA scales.

Additional notes

Additional markers of the type 'boundary' can be found in all described tasks and files. Events of such type are added automatically by EEGLAB when some portions of the data were deleted, or when portion of continuous datasets are concatenated. Of note, this occurs at the beginning of each run after uploading the data from Brain Products format to EEGLAB. Some raw files were concatenated because of technical errors during recordings, which required saving more than one file for each task. In these files, when a new portion of data was introduced, an additional marker with the code 'New Segment' was created. Files that were prepared that way are as follows: sub-17 (MSIT), sub-21 (MSIT), sub-23 (MSIT), sub-25 (REST), sub-37 (oddball), sub38 (oddball, MSIT). Furthermore, one participant did not complete the SRT task correctly (sub_29).

Reliability

Methods

Behavioral data for the SRT and oddball task were calculated from EEG files (i.e., ‘*_events.tsv’) and MSIT+ data were calculated from original Presentation (*.log) files. The raw files were used because these provide more precise information about individual stimuli. Trials that were missed (i.e., no responses), anticipation (faster than 100 ms for SRT/oddball task and 200 ms for MSIT task), and longer than the time of stimulus plus the shortest used ITI were coded as errors for all tasks. Additionally, when participant had multiple responses for one stimulus, this was also coded as an error in MSIT task. One participant was excluded in the calculation of behavioral data for the SRT task, as his/her responses were not recorded correctly (sub_29).

Statistical analysis of RTs was performed in JASP (version 0.14.1.0) [27]. Repeated Measures ANOVA was conducted with task conditions as repeated measures factors and Shapiro-Wilk test was used to test normal distribution. The assumption of sphericity was violated and Greenhouse-Geisser correction was used.

Dataset quality

We placed great emphasis on obtaining clean EEG data. Electrode-skin impedance was kept as low as possible (average ± SD impedance = 4.67 ± 2.33 kΩ; Tab. 7), and participants were informed about the deteriorating influence of their movements on the EEG quality. We observed recorded EEG signals via remote desktop connection and intervened when necessary. Unavoidably, there are eye-blink artifacts on frontal electrodes, and often muscle artifacts on temporal and occipital poles. A large signal drift is also observed for some participants/electrodes as no high-pass filters were used (this step was omitted to ensure the possibility of further data modifications/filtering according to analytical needs).

Data preprocessing for the purpose of our own analyses (not included in the database) indicated relatively good quality of EEG signal. Indeed, on average, in the most restrictive and conservative approach, we excluded 22.1 ± 14% of trials.

Table 7. Average impedance levels for the MSIT task, for each participant.

**Subject ID**	**Impedance levels [kΩ]**
	Mean
sub-01	4.21
sub-02	3.63
sub-03	4.95
sub-04	3.70
sub-05	6.09
sub-06	4.73
sub-07	3.98
sub-08	4.78
sub-09	3.93
sub-10	5.07
sub-11	6.00
sub-12	5.19
sub-13	5.43
sub-14	2.64
sub-15	4.20
sub-16	2.50
sub-17	3.51
sub-18	3.93
sub-19	2.18
sub-20	3.48
sub-21	3.30
sub-22	4.47
sub-23	8.71
sub-24	11.49
sub-25	3.48
sub-26	2.57
sub-27	4.61
sub-28	3.03
sub-29	5.52
sub-30	5.04
sub-31	3.80
sub-32	15.01
sub-33	5.12
sub-34	4.89
sub-35	4.24
sub-36	4.13
sub-37	2.92

sub-38	3.89
sub-39	2.92
sub-40	6.27
sub-41	3.89
sub-42	2.91

*Based on MSIT+ files. The exact data of impedance during MSIT+ task for sub-06 were not available. The impedances recorded during the oddball task are included, in turn.

Dataset basic results

The basic behavioral and ERP results also confirm the quality of this dataset. Indeed, reaction times and accuracy corresponded with the increasing difficulty of tasks conditions, as expected. Further, typical ERP waves were obtained, as expected, for attention and cognitive control tasks (e.g., N200, P300, N450).

Responses in SRTs are usually faster than responses in any choice reaction tasks. As expected, our mean SRT latencies were fast (Tab. 8 and Fig. 4), and were faster than observed in the 00 (no conflict) MSIT condition, and as compared to oddball responses. In addition, participants made very few errors: 4.23%, including 2.12% related to response failures and only 1.9% related to premature responses (i.e., faster than 100 ms). The rest of the errors corresponded with late/long responses.

[Figure 4.]

Fig. 4. Reaction time data for SRT and MSIT tasks

Tab. 8 summarizes the behavioral data. Three participants most likely misunderstood the oddball instruction and frequently responded to distractor/deviant stimuli (sub_02 – false alarm to distractors = 98.77%; sub_14 – false alarm to distractor = 97.53%; sub_17 – false alarm to distractors = 76.54%). These three participants were excluded from the behavioral summary of oddball task provided in Tab. 8. Stimuli similarity in the oddball paradigm made the task quite

difficult for participants, as they committed on average 9.52 ± 8.90% of errors towards distractors.

Table 8. Summary of behavioral results

	Participants (SRT: N = 41; Oddball: N = 39; MSIT: N = 42)	
	Mean	SD
**SRT**		
Reaction time to stimulus (ms)	294.69	43.28
**Oddball**		
Reaction time to targets (ms)	542.13	65.50
Target omission (%)	5.60	8.33
False alarm to distractors (%)	9.52	8.90
False alarm to standards (%)	0.24	0.46
**MSIT+**		
Reaction time to 00 (ms)	537.29	62.11
Reaction time to S0 (ms)	592.61	54.48
Reaction time to F0 (ms)	678.71	69.80
Reaction time to FS (ms)	746.82	85.87
00 response errors (%)	0.90	2.85
S0 response errors (%)	0.69	2.22
F0 response errors (%)	1.23	3.55
FS response errors (%)	1.55	3.83

The gradual increase of the conflict over the four conditions of the MSIT+ is clearly seen in reaction times (00<S0<F0<FS, Fig. 4; $F(1.76, 72.02) = 391.15$, $p < 0.01$; all post-hoc pairwise comparisons significant with $p < 0.01$ and Bonferroni correction). As expected, more response errors were observed for more demanding conditions, for example, more errors were observed for F0 (1.23 ± 3.55%) and FS (1.55 ± 3.83%) as compared to 00 (0.90 ± 2.85%) and S0 (0.69 ± 2.22%) conditions. However, 16 out of 42 participants (38%) did not commit any errors, and another 19 committed 1-3 errors during the entire task. Therefore, a total of 83% of participants did not commit more than 3 errors during the whole task. Only four participants committed

substantially more errors during the task (>17). Given these observations, more elaborate error-rate analyses are not possible nor recommended.

Dataset ERP results

The aim of this section was to show basic characteristics of the obtained signal. During both the MSIT+ and oddball tasks, the primary ERPs are clearly visible (Fig. 5).

[Figure 5.]

Fig. 5. Grand averaged ERP (with standard deviation) for oddball and MSIT+ tasks, shown with two distinct midline electrodes Fz (more frontal pole) and Pz (parietal pole)

The most studied component in healthy and clinical populations is P3, which peaks between 300-600 ms after stimulus onset [28]. P3 closely reflects attentional and memory processes in the human brain [28], and is often studied in the context of an oddball task. A much higher amplitude of P3 induced by target in comparison to distractor stimuli, and standard trials are visible in our oddball task results, especially for electrodes placed in parietal areas (such as Pz), which agrees with the current literature [29]. Additionally, there is a clear distinction between two P3 subcomponents — namely P3a and P3b — in our 3-stimulus oddball task. The P3a is more likely to be evoked during tasks with novelty, whereas P3b is considered to reflect attention [29]. Another well studied component related to cognitive control, attention, and conflict resolution is the fronto-central negative peak (N2). The N2 is commonly observed between 200-350 ms after stimulus onset [30]. A clear fronto-central N2 is observed in our data collected during the oddball task (Fig. 5). In MSIT+ ERPs, a later conflict-related negativity (i.e., the N450) is observed on fronto-central electrodes (Fig. 5). Of note, the N450 extends more posterior as a negative deflection within the P3. The P3b and N450 clearly discriminate the four conditions of MSIT task. In particular, the P3 shows higher amplitudes for faster

responses (00>S0>F0>FS) whereas the N450 amplitude increases with increasing levels of conflict (00<S0<F0<FS). Yet another conflict-related wave is clearly visible in our MSIT data: the slow potential (SP). The SP is commonly observed after a crossover point on the descending slope of a waveform [31]. The conflict SP amplitude increases with increasing levels of conflict (00<S0<F0<FS), as expected from earlier literature [32]. These basic results show that the tasks were designed correctly.

Summary and perspectives

It is essential to integrate and re-use data to improve the reliability of results in neuroscience. Our Nencki-Symfonia EEG dataset is well suited for neuropsychological research regarding common and distinct mechanisms involved in different cognitive tasks. A wide range of classical EEG/ERP features can be compared across tasks for any chosen subset of electrodes. At the same time, full high-density EEG recording allows for source localization and detailed connectivity studies. Neurophysiological measures can be also correlated with the psychometric data. This dataset can also be used to develop and verify novel analytical and classification approaches, including advances in the field of deep/machine learning algorithms, recognition of single-trial ERP responses to different task conditions, and detection of particular EEG/ERP features for use in BCI applications. BCI as a discipline could be placed between neuroscience and computer science, and brings new solutions for the disabled or ill, as well as for healthy individuals. Most studies tend to focus on two main solutions: a) BCIs based on movement control for preparation of robotic limbs with need of motor imagery (MI) tasks, and b) attentional based BCIs used to create direct communication systems which are often based on steady-state visual evoked potential (SSVEP). However, these paradigms may not always work for all patients, especially those in complete locked-in syndrome (i.e., individuals who do not

exhibit eye movements). Further, there is a need to modernize our approach to BCIs and develop gaze-independent approaches. Active eye movements are not necessary when stimuli are presented sequentially. For example, the P300 component is elicited by stimuli that draw participant attention and is frequently used for BCI control [33, 34]. The development of improved BCI requires a deeper understanding of the basics of EEG signals, functioning of brain areas and the connections between them, with a strong emphasis on attention control. Some approaches have attempted to use conflict tasks (e.g., semantically congruent and incongruent stimuli) to first, evaluate performance, and then, build BCI systems for patients with disorders of consciousness (DOC) [34]. As seen above, BCIs still have many challenges that could be addressed with a more robust system training that would allow better accuracy. A more robust system would also improve the ability of the system to learn to 'read' real brain signals between nonstationary noises and artifacts.

Deep/machine learning approaches are widely used to detect specific patterns of EEG activity and improve understanding of brain functions [35, 36, 37, 38, 39]. These approaches are promising for developing new medical methodologies for early intervention and treatment of various brain dysfunctions, such as depression, stress-induced conditions, Alzheimer's disease, autism spectrum disorder, attention deficit hyperactivity, and more [40]. Neural Attention Models are the most recent state-of-the-art deep learning approaches that show promise in this area [41]. There is also a critical need to improve feature extraction, i.e., the process of analyzing signals to distinguish signal features from extraneous content. The proposed dataset could serve as a benchmark and help to evaluate the performance of several novel classifiers in an off-line scenario. Such a process is frequently used to evaluate new approaches (cf. [42]).

Advances in the ability to decode mental and cognitive states is a vital branch of neuroscience and computer science that has gained new attention in recent years [43, 44, 45, 46]. Approaches based on machine learning can decode task engagement, performance, and attention from MSIT

and oddball tasks, which can be vital for BCI and novel systems that could detect early mental fatigue. This could be used as a biomarker of fatigue, especially in professions where there is a need for constant attention (e.g., air traffic controllers, professional drivers). Resting-state brain dynamics have been shown to predict the effects of oddball paradigms. For example, rest regional power of a few brainwaves have been shown to correlate with the latency and amplitude of P300, N3, P2, and N1 components [47].

All the tasks included in this dataset can also be used for the in-depth study of the so-called "time-on-task" (time spent to solve the problem) problem [48]. The "time-on-task" concept, in part, appears to oppose the results of conflict processing studies. For example, it has been asserted that the dorsal medial frontal cortex is more sensitive to time-on-task rather than conflict itself.

Summary of the advantages of this dataset

- localizer data of individual electrode locations, which can greatly improve the reliability of EEG source analysis given that real electrode localization can slightly vary from standard templates and across participants due to differences in head geometry;
- recorded A1 and A2, which enables the off-line possibility of using classical ear-referenced montages;
- high-density recording, which allows for in-depth connectivity and source localization studies;
- 4 different tasks completed by the same participants;
- the first publicly available dataset that includes the MSIT in healthy participants;
- corresponding resting-state questionnaire along with EEG resting-state data;
- relatively large sample size (N = 42);

- twofold verification of the data via behavioral and simple ERP investigation;
- detailed health data about participants (menstrual cycle phase, medication & caffeine use).
-

Availability of supporting data

All the data described in this paper, including EEG datasets, behavioral, and basic questionnaire data, are available in The Nencki-Symfonia EEG/ERP dataset repository [GigaScience database, GigaDB, *cite unique persistent identifier*].

Abbreviations

EEG: electroencephalography; ERP: event-related potentials; BCI: brain-computer interface; MSIT+: extended Multi-Source Interference task. SRT: Simple reaction task. REST: resting-state, ARSQ: Amsterdam resting-state questionnaire, UWIST: Mood Adjective Check List (UMACL).

Ethics, consent and permissions

This study was performed in accordance with the declaration of Helsinki and was approved by the Research Ethics Committee, Faculty of Humanities, Nicolaus Copernicus University in Toruń, Poland (No. 6/2018). All participants provided written informed consent to participate in the study and received cash remuneration (200 PLN, around 44 EUR).

Acknowledgments

This work was supported by Polish National Science Centre (NCN) grant no. 2016/20/W/NZ4/00354.

Competing interests

The authors declare that they do not have any competing interests.

Authors' contributions

All authors designed the MSIT & SRT experiments, JD & JW designed the oddball experiment, JD prepared the Polish version of the ARSQ, JW implemented the experiments, PD & IA acquired the data, PD & IA performed data analysis, PD wrote first manuscript and IA, JD, & EK reviewed the manuscript, all authors read/accept the final manuscript, EK supervised the entire study.

References

[1] Teeters JL, Harris KD, Millman KJ, Olshausen BA, Sommer FT. Data sharing for computational neuroscience. Neuroinformatics. 2008;6(1):47-55.

[2] Wiener M, Sommer FT, Ives ZG, Poldrack RA, Litt B. Enabling an Open Data Ecosystem for the Neurosciences. Neuron. 2016;92(3):617-621. Erratum in: Neuron. 2016;92(4):929.

[3] Ascoli GA, Maraver P, Nanda S, Polavaram S, Armañanzas R. Win-win data sharing in neuroscience. Nat Methods. 2017;14(2):112-116.

[4] White T, Blok E, Calhoun VD. Data sharing and privacy issues in neuroimaging research: Opportunities, obstacles, challenges, and monsters under the bed. Hum Brain Mapp. 2020;10.1002/hbm.25120.

[5] De Jong R, Liang CC, Lauber E. Conditional and unconditional automaticity: a dual-process model of effects of spatial stimulus-response correspondence. J Exp Psychol Hum Percept Perform. 1994;20(4):731-50.

[6] Egner T, Delano M, Hirsch J. Separate conflict-specific cognitive control mechanisms in the human brain. Neuroimage. 2007;35(2):940-8.

[7] Hommel B. Theory of Event Coding (TEC) V2.0: Representing and controlling perception and action. Atten Percept Psychophys. 2019;81(7):2139-2154.

[8] Carter CS, van Veen V. Anterior cingulate cortex and conflict detection: an update of theory and data. Cogn Affect Behav Neurosci. 2007;7(4):367-79.

[9] Coderre E, Conklin K, van Heuven WJ. Electrophysiological measures of conflict detection and resolution in the Stroop task. Brain Res. 2011;1413:51-9.

[10] Jiang J, Zhang Q, Van Gaal S. EEG neural oscillatory dynamics reveal semantic and response conflict at difference levels of conflict awareness. Sci Rep. 2015;5:12008.

[11] Eriksen BA, Eriksen CW. Effects of noise letters upon the identification of a target letter in a nonsearch task. Perception & psychophysics. 1974;16(1):143-9.

[12] Simon JR, & Wolf JD. Choice reaction time as a function of angular stimulus-response correspondence and age. Ergonomics 1963;6(1), 99–105.

[13] Bush G, Shin LM, Holmes J, Rosen BR, Vogt BA. The Multi-Source Interference Task: validation study with fMRI in individual subjects. Mol Psychiatry. 2003;8(1):60-70.

[14] Bush G, Shin LM. The Multi-Source Interference Task: an fMRI task that reliably activates the cingulo-frontal-parietal cognitive/attention network. Nat Protoc. 2006;1(1):308-13.

[15] Basu I. Intracranial EEG dataset 1 [Dataset]. Zenodo. 2021. https://doi.org/10.5281/zenodo.5083120

[16] Basu I. Intracranial EEG dataset 2 [Dataset]. Zenodo. 2021. https://doi.org/10.5281/zenodo.5085197

[17] Widge A & Zorowitz S. Effects of ON/OFF deep brain stimulation on cognitive control in treatment-resistant depression (EEG). OpenNeuro [Dataset]. 2019.

[18] Widge AS, Zorowitz S, Basu I, Paulk AC, Cash SS, Eskandar EN, Deckersbach T, Miller EK, Dougherty DD. Deep brain stimulation of the internal capsule enhances human cognitive control and prefrontal cortex function. Nat Commun. 2019;10(1):1536.

[19] Oldfield RC. The assessment and analysis of handedness: the Edinburgh inventory. Neuropsychologia. 1971;9(1):97-113.

[20] Goryńska E. The mood adjective checklist (UMACL) by Matthews, chamberlain and Jones. Warsaw: Polskie Towarzystwo Psychologiczne. Poland. 2005.

[21] Matthews G, Jones DM, Chamberlain AG. Refining the measurement of mood: The UWIST mood adjective checklist. British journal of psychology. 1990;81(1):17-42.

[22] Diaz BA, Van Der Sluis S, Moens S, Benjamins JS, Migliorati F, Stoffers D, Den Braber A, Poil SS, Hardstone R, Van't Ent D, Boomsma DI, De Geus E, Mansvelder HD, Van Someren EJ, Linkenkaer-Hansen K. The Amsterdam Resting-State Questionnaire reveals multiple phenotypes of resting-state cognition. Front Hum Neurosci. 2013;7:446.

[23] Wiesman AI, Wilson TW. Posterior Alpha and Gamma Oscillations Index Divergent and Superadditive Effects of Cognitive Interference. Cereb Cortex. 2020;30(3):1931-1945.

[24] Gorgolewski KJ, Auer T, Calhoun VD, Craddock RC, Das S, Duff EP, Flandin G, Ghosh SS, Glatard T, Halchenko YO, Handwerker DA, Hanke M, Keator D, Li X, Michael Z, Maumet C, Nichols BN, Nichols TE, Pellman J, Poline JB, Rokem A, Schaefer G, Sochat V, Triplett W, Turner JA, Varoquaux G, Poldrack RA. The brain imaging data structure, a format for organizing and describing outputs of neuroimaging experiments. Sci Data. 2016;3:160044.

[25] Pernet CR, Appelhoff S, Gorgolewski KJ, Flandin G, Phillips C, Delorme A, Oostenveld R. EEG-BIDS, an extension to the brain imaging data structure for electroencephalography. Sci Data. 2019;6(1):103.

[26] Delorme A & Makeig S. EEGLAB: an open-source toolbox for analysis of single-trial EEG dynamics, Journal of Neuroscience Methods 2004;134:9-21.

[27] JASP Team (2021). JASP (0.14.1.0) [Computer software].

[28] Polich J, Herbst KL. P300 as a clinical assay: rationale, evaluation, and findings. Int J Psychophysiol. 2000;38(1):3-19.

[29] Polich J. Updating P300: an integrative theory of P3a and P3b. Clin Neurophysiol. 2007;118(10):2128-48.

[30] Folstein JR, Van Petten C. Influence of cognitive control and mismatch on the N2 component of the ERP: a review. Psychophysiology. 2008;45(1):152-70.

[31] West R, Jakubek K, Wymbs N, Perry M, Moore K. Neural correlates of conflict processing. Exp Brain Res, 2005;167:38-48.

[32] Larson MJ, Clayson PE, Clawson A. Making sense of all the conflict: A theoretical review and critique of conflict-related ERPs. International Journal of Psychophysiology, 2014;93:283-297.

[33] Farwell LA, Donchin E. Talking off the top of your head: toward a mental prosthesis utilizing event-related brain potentials. Electroencephalogr Clin Neurophysiol. 1988;70(6):510-523.

[34] Xie Q, Pan J, Chen Y, et al. A gaze-independent audiovisual brain-computer Interface for detecting awareness of patients with disorders of consciousness. BMC Neurol. 2018;18(1):144.

[35] Marblestone AH, Wayne G, Kording KP. Toward an Integration of Deep Learning and Neuroscience. Front Comput Neurosci. 2016;10:94.

[36] Vieira S, Pinaya WH, Mechelli A. Using deep learning to investigate the neuroimaging correlates of psychiatric and neurological disorders: Methods and applications. Neurosci Biobehav Rev. 2017;74(Pt A):58-75.

[37] Vu MT, Adalı T, Ba D, Buzsáki G, Carlson D, Heller K, Liston C, Rudin C, Sohal VS, Widge AS, Mayberg HS, Sapiro G, Dzirasa K. A Shared Vision for Machine Learning in Neuroscience. J Neurosci. 2018;38(7):1601-1607.

[38] Lindsay GW. Attention in Psychology, Neuroscience, and Machine Learning. Front Comput Neurosci. 2020;14:29.

[39] Vahid A, Mückschel M, Stober S, Stock AK, Beste C. Applying deep learning to single-trial EEG data provides evidence for complementary theories on action control. Commun Biol. 2020;3(1):112.

[40] Valliani AA, Ranti D, Oermann EK. Deep Learning and Neurology: A Systematic Review. Neurol Ther. 2019 Dec;8(2):351-365.

[41] de Santana Correia A, Colombini EL. Attention, please! A survey of Neural Attention Models in Deep Learning. arXiv preprint arXiv:2103.16775. 2021.

[42] AlZoubi O, Koprinska I, Calvo RA. Classification of Brain-Computer Interface Data. Proceedings of the 7th Australasian Data Mining Conference - Volume 87 (AusDM '08). Australian Computer Society, Inc.. 2008(87), 123–131.

[43] Jiang J, Heller K, Egner T. Bayesian modeling of flexible cognitive control. Neurosci Biobehav Rev. 2014;46 Pt 1:30-43.

[44] Hawkins GE, Mittner M, Boekel W, Heathcote A, Forstmann BU. Toward a model-based cognitive neuroscience of mind wandering. Neuroscience. 2015;310:290-305.

[45] Provenza NR, Paulk AC, Peled N, Restrepo MI, Cash SS, Dougherty DD, Eskandar EN, Borton DA, Widge AS. Decoding task engagement from distributed network electrophysiology in humans. J Neural Eng. 2019;16(5):056015.

[46] Yousefi A, Basu I, Paulk AC, Peled N, Eskandar EN, Dougherty DD, Cash SS, Widge AS, Eden UT. Decoding Hidden Cognitive States From Behavior and Physiology Using a Bayesian Approach. Neural Comput. 2019;31(9):1751-1788.

[47] Lee TW, Yu YW, Wu HC, Chen TJ. Do resting brain dynamics predict oddball evoked-potential? BMC Neurosci. 2011;12:121.

[48] Grinband J, Savitskaya J, Wager TD, Teichert T, Ferrera VP, Hirsch J. The dorsal medial frontal cortex is sensitive to time on task, not response conflict or error likelihood. Neuroimage. 2011;57(2):303-11.

Figure1 Click here to access/download;Figure;Figure1_tiff_600dpi_85mm_half_page.tif

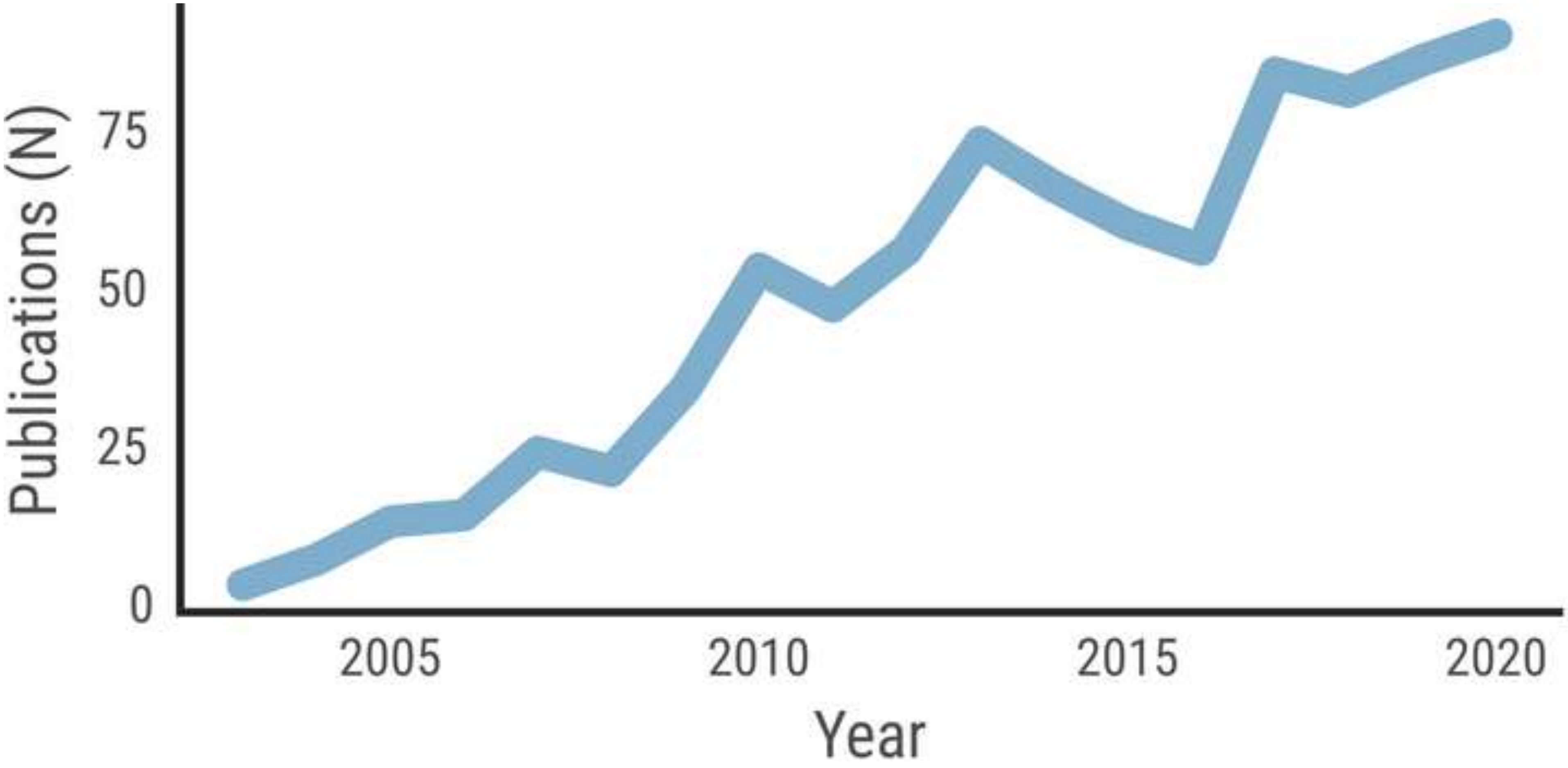

Figure2

Click here to access/download;Figure;Figure2_tiff_600dpi_170mm_full_page.tif

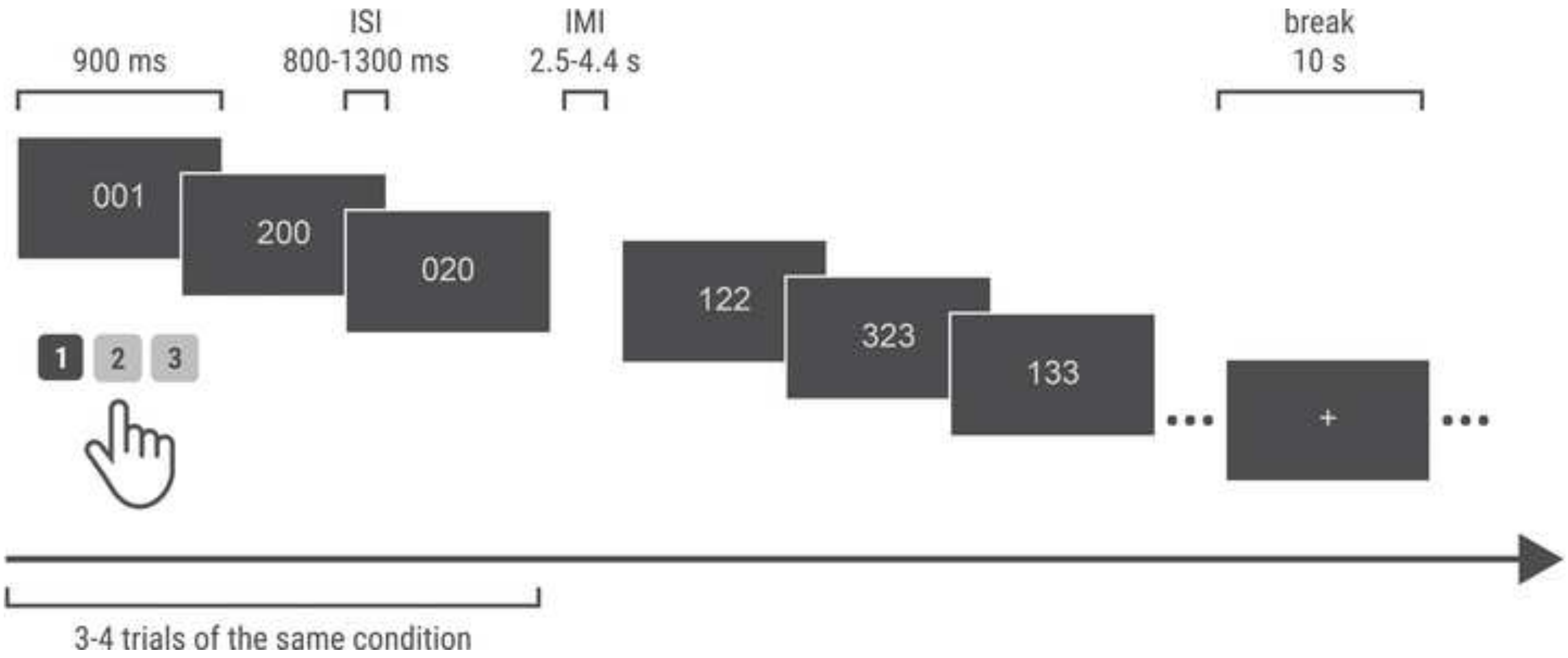

Figure3

Click here to access/download;Figure;Figure3_tiff_600dpi_170mm_full_page.tif

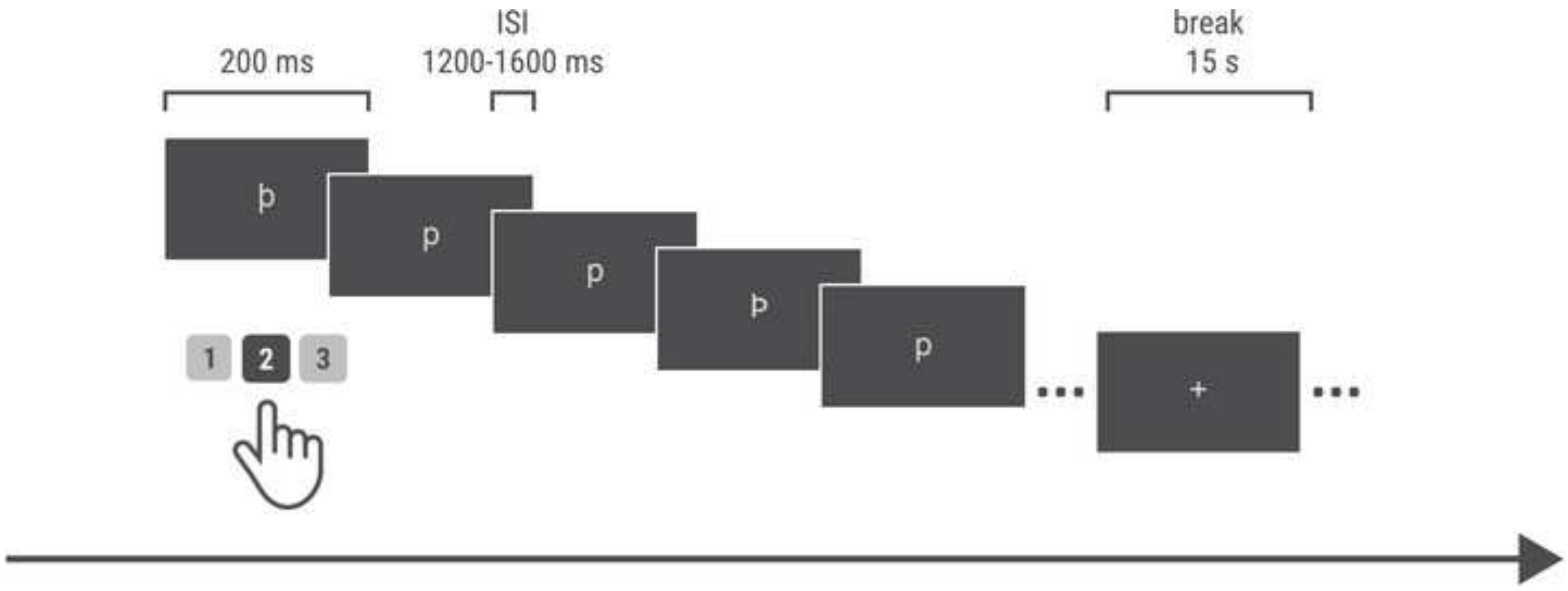

Figure4

Click here to access/download;Figure;Figure4_tiff_600dpi_85mm_half_page.tif

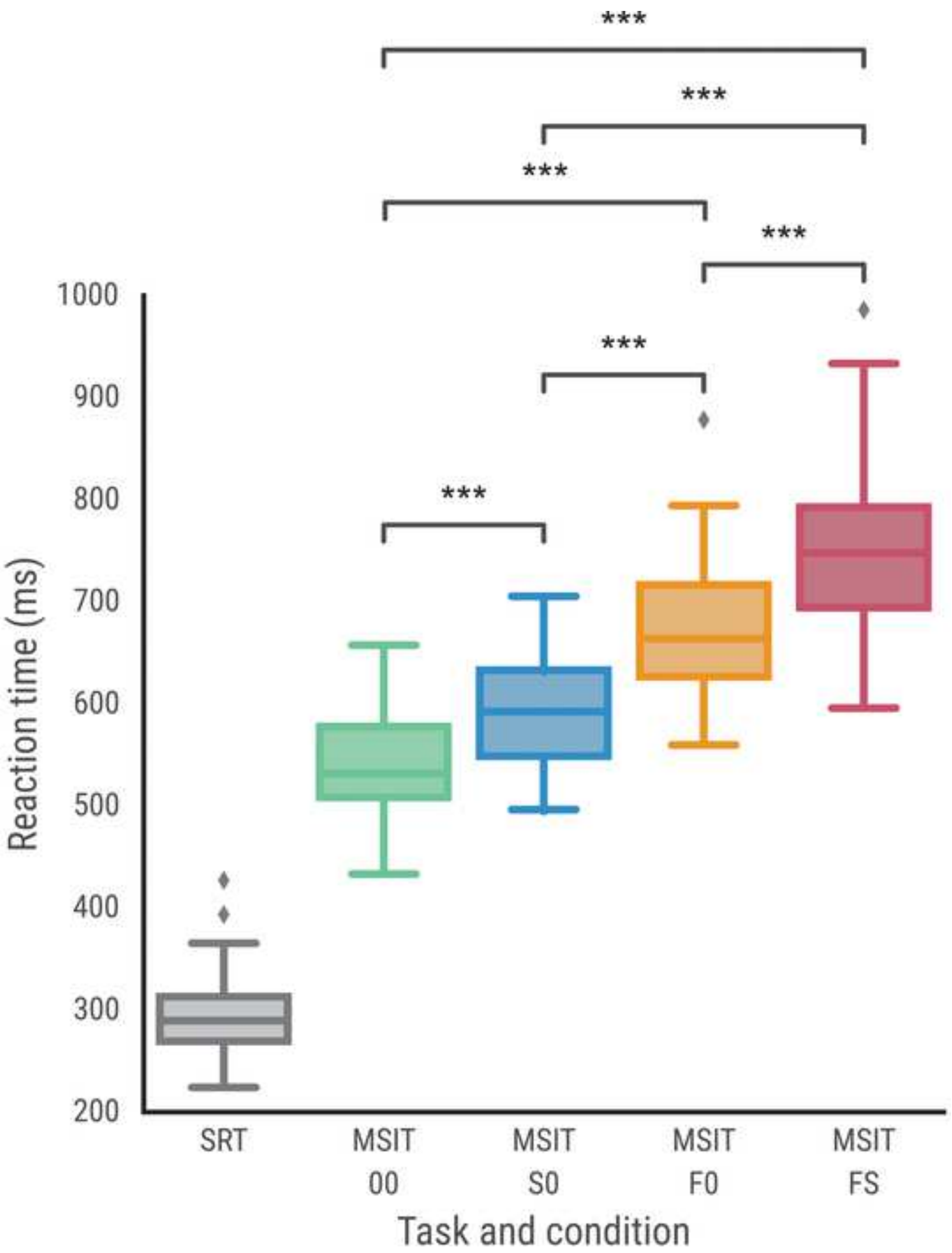

Figure5

Click here to access/download;Figure;Figure5_tiff_600dpi_170mm_full_page.ti

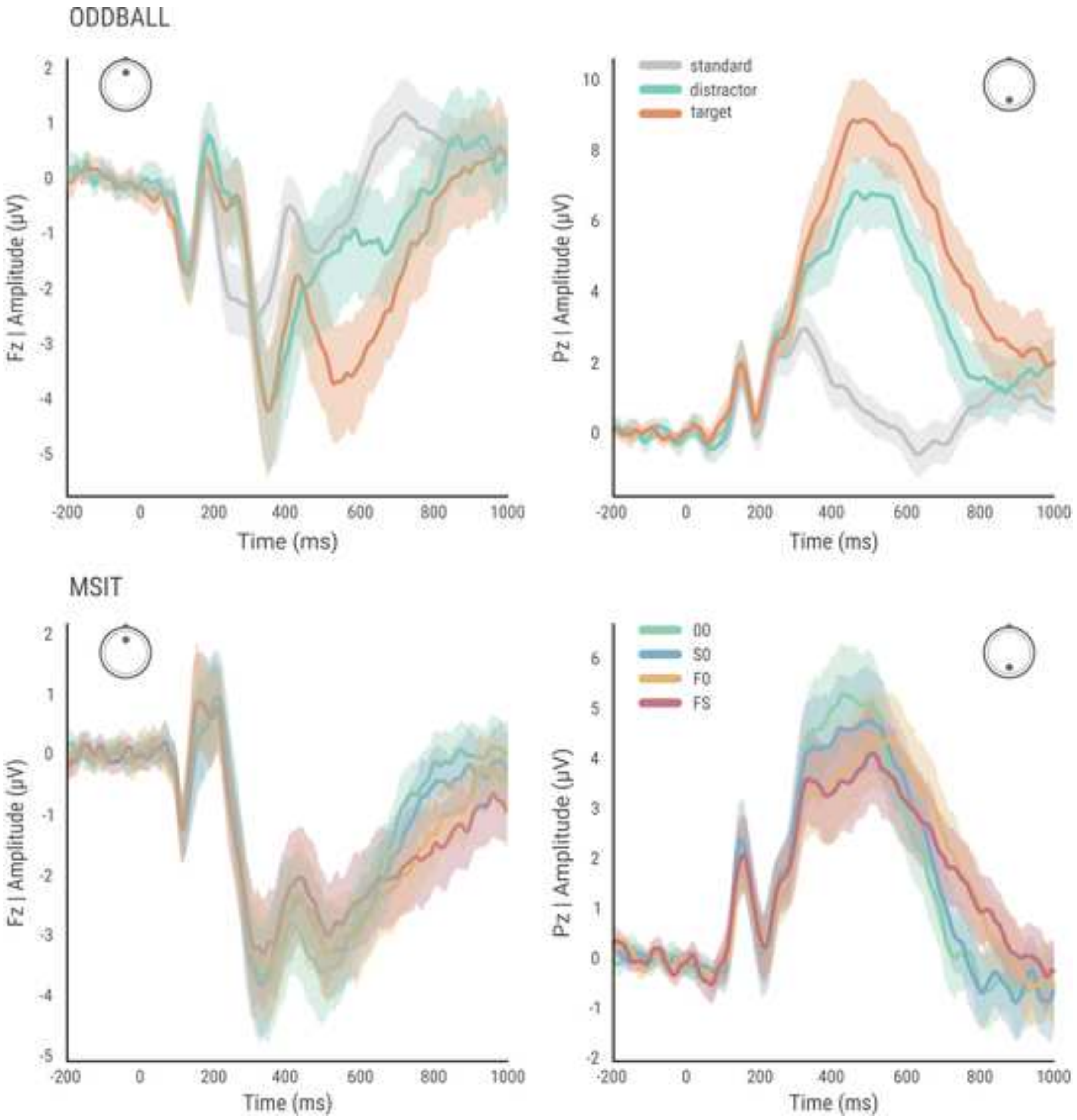

Supplement: giac015_GIGA-D-21-00298_Original_Submission [file giac015_giga-d-21-00298_original_submission.pdf]
