# Supplementary material for: The Nencki-Symfonia electroencephalography/event-related potential dataset: Multiple cognitive tasks and resting-state data collected in a sample of healthy adults
Source: Gigascience. 2022 Mar 7;11:giac015. doi: 10.1093/gigascience/giac015 (PMC8900497; doi:10.1093/gigascience/giac015)
Supplement: giac015_Response_to_Reviewer_Comments_Revision_1 [file giac015_response_to_reviewer_comments_revision_1.pdf]

Author's Response To Reviewer Comments

Close

Thank you very much for the opportunity to prepare the revised version of our manuscript. We have carefully read all the precise and constructive suggestions and we tried to answer the reviewers' concerns. We believe that our manuscript is now improved and appreciate the time and effort the reviewers and you have dedicated to evaluate our manuscript.

Here, we attached our responses to the reviewers' comments. The revised manuscript is uploaded to the GigaScience website with changes in text marked with red font color (www.editorialmanager.com/giga/).

Our manuscript describes dataset only, we do not have any software application to declare and register in the indicated databases.

We have changed the section name from 'Acknowledgments' to 'Funding' and we declared additional information about our funding status and PI.

We have also left the information "cite unique persistent identifier" in the "Availability of supporting data" section, as we do not know the identifier of our GigaDB.

We would like to add additional corresponding author (Ewa Kublik), which we marked in the manuscript. However, we did not mark that during submission to maintain one person responsible for submission process (error-note from editorialmanager.com/giga/ site: "PLEASE NOTE: If you continue with this author as the Corresponding Author, then once you build the PDF or exit the submission process, you will no longer have access to the submission. This author must then log into the system and approve the PDF to complete the submission of your manuscript.").

Response to Reviewer #1:
Thank you very much for your valuable feedback and insightful comments regarding our manuscript. We revised and modified our manuscript accordingly to your comments. More details and complete responses are added below:

Response #1: We have rephrased the second sentence to make it simple and straight-forward.
As for the 'resting state task' we replaced the word 'task' with the word 'protocol' and changed the beginning of the mentioned sentence.

Response #2: In our experiments we were focused on the visual domain, but in the introduction we should of course also include more general information. We have added the reference pointing to an article describing conflict resolution during two Simon tasks with additional task-irrelevant tactile or auditory information (Mahani et al. 2019).

Response #3: As suggested, we added a short paragraph 'Summary of the limitations of this dataset', including reservations indicated by the Reviewer.
As for the comment #3a, we fully agree that our EEG dataset cannot be directly compared with fMRI results presented in the literature. Our original plan was to obtain high density EEG and fMRI data from the same task and the same participant, which would make the two sets of results as close to each other as possible. All the data was acquired but now the MRI set cannot be openly accessed because of the inner regulation of our consortium partner, who has the main authorship of MRI data. The data can be obtained on the individual request.
We would also like to stress that our EEG recording with individual electrodes' location data can be used for reliable source analyses, which can be cautiously cross-referenced with the fMRI results.
As for comment #3b. Stimulus and ISI durations are similar to those used by Bush et al. 2006, to study healthy populations (ITI was fixed there at 1750 ms, both containing stimulus and ISI, 24 trials were displayed in 42 s blocks). We added pseudorandom component to ISIs to reduce the likelihood of

anticipating the onset of the stimulus (the slowest ISI in our case was 800 ms, and the longest 1300 ms; ITI 1700-2200 ms). Given the average response times from 537.29 ms to 746.82 ms, there is still ~1 s sweep after the movement termination (less than 1 s in FS condition with longest RT, and more than 1 s in all other trials). Although, we agree that this experimental setup could not be used to study oscillatory (e.g. motor related) dynamics over 1.5-2 seconds. We have added that information to the text with corresponding literature sources.

Response #4: We deleted the sentence where the ‘extended psychometric’ phrase appeared. It was our initial idea to include the information about existing additional data in the manuscript (i.e. corresponding MRI T1 and extended psychometric data; that are not included in the dataset, but available for the readers upon request). However, this is not in line with the journal's requirements (Submission Guidelines), and we were asked to delete the information about the data which cannot be uploaded along with our dataset. The other data belong to our consortium partners and for now, due to the internal regulations of those institutes, we are still not eligible to publish them openly.

Response #5: The experimental group was highly homogeneous in regard to the education and social status, the majority of the participants were students. That was a highly educated group. We have added this general information to the manuscript. Although, we do not have exact information about years of completed education (only for 50% of the subjects).

Response #6: The Polish adaptation of ARSQ belongs to the consortium partner (who also owns the extended psychometric data), who plans to publish extended analyses regarding the reliability and integrity of the Polish version of ARSQ in a separate, dedicated article.

Response #7: The original paper about MSIT (Bush et al. 2006) task proposes that the training should last 1-5 min and we followed this suggestion. We have added the extended description of the training tasks hopefully answering your questions:
‘Training sessions of MSIT+ and oddball tasks were performed by each participant before the main task execution. In case of both tasks, ~45-50 stimuli were presented in a training session. The MSIT+ training lasted for ~2.5 min., whereas oddball training lasted ~1.3 min. One training session was sufficient for most subjects. However, if more errors were made, then the participant went through the training again until the instructions were completely understood.’

Response #8: As mentioned above, for their fMRI study Bush et al. (2006) used block design with 24 trials of the same category displayed in a row. Such design generally yields higher statistical power than fast event-related design in fMRI. We decided to propose a more universal paradigm, which would partially retain the benefits of blocked stimulation but also allow for event-related analysis in both EEG and fMRI experiments. As can be seen in the submitted manuscript, our MSIT mini-block design has the same general pattern in behavioral results, as block design (Bush et al. 2006) or event-related design used by other groups (Wiesman et al. 2020).

Response #9: Thank you very much for this hint, we have corrected the text.

Response #10: There is one peer-reviewed proceedings paper related to machine-learning on these data (MSIT, SRT) that was presented during IEEE Symposium on Bioinformatics and Bioengineering (BIBE). It just got published with a DOI 10.1109/BIBE52308.2021.9635187. We added it to the reference list and mentioned it at the end of a paragraph ‘Dataset ERP results’. Other research manuscripts are now under peer-review process.
Dzianok, P., Kołodziej, M., & Kublik, E. (2021). Detecting attention in Hilbert-transformed EEG brain signals from simple-reaction and choice-reaction cognitive tasks. In 2021 IEEE 21st International Conference on Bioinformatics and Bioengineering (BIBE) (pp. 1–4). DOI:10.1109/BIBE52308.2021.9635187

Response #11: As suggested, we re-calculated average impedances for other tasks (and resting state) and added them to Table 4. `

Response #12: We believe that standard deviation corridors presented in the figure clearly illustrate the waveform differences, particularly strong in the odd-ball task. It is less obvious for the four MSIT+ categories, for which we found complex interaction effects not easy to illustrate in one simple figure. This data was extensively analyzed with ERP-microstates approach and cluster-permutation waveform analysis and obtained results will be soon published.

Response #13: No fixation point was presented, the fixation cross '+' was presented only during the breaks that are marked on the drawings. Blank black screen appeared between stimuli. We added such information to the manuscript now.

Response #14: We have added the swarm plots to each boxplot, showing individual data in a concise and readable way.

Response to Reviewer #2
Thank you very much for your time dedicated to reviewing our manuscript and offering helpful remarks. The answers for your questions are given below:

Response #1: We have added the following information to the manuscript: 'Signal artifacts (eye-movements, cardiac, muscle artifacts) were corrected for the purpose of ERP plotting using Independent Component Analysis (ICA; [28]). Remaining artifacts were removed during semi-automatic visual inspection. Data was band-pass filtered (0.5-40 Hz), re-referenced to an average reference and segmented into epochs from 200 ms before stimulus presentation to 1000 ms after stimulus presentation. Clean segments with correct responses were averaged separately for every condition and task. Baseline correction was applied for visualization purposes.'

Response #2: Thank you for that suggestion, we have added the oddball reaction time to Figure 4.

Response #3: The second time slot was only four hours after the first one. That was still early afternoon (1 PM), to ensure that our participants will be fully rested and perform the tasks with the utmost care. Some participants preferred the second time slot, as the first time slot was too early for their daily routine (9 AM). We did not force participants for one of the time slots, there was always a choice between the morning one and the early-afternoon one. We added one additional sentence for that matter in the manuscript.

Response #4: Tasks were divided into two or four runes (all were recorded) according to Table 2., and for ease of downloading and processing the data, all runes for each task are saved in one file (per participant). I hope this answers your question. Additionally, we changed the third column name from 'Runs [no.]' to 'Recorded runs [no.] in Table 2.

Response #5: On average, 399±6 [M±SD] trials were presented to each subject (average number of trials for each condition – 00: 102±7 [M±SD], S0: 105±7 [M±SD], F0: 94±9 [M±SD], FS: 98±9 [M±SD]). We have added that information to the manuscript.

Response #6: Thank you for this comment. You are right, Tab. 4-6 were deleted during the edition process and merged into Tab. 3. and we unfortunately overlooked the uncorrected numbering of the following tables. The numbering of the tables and their referencing in the text were corrected and checked carefully.
As for the location of the impedance table in the text (and consequently its number), we prefer to leave it as it was – in the 'Dataset quality' subsection, where it well illustrates the thoroughness of preparing the setup for each recording. In the method section we provided a reference to this table, to draw the reader's attention to the fact that all the resistance data are given further in the text. However, we do not think that this table should be put so early in the text.

Close
