# Supplementary material for: The Nencki-Symfonia electroencephalography/event-related potential dataset: Multiple cognitive tasks and resting-state data collected in a sample of healthy adults
Source: Gigascience. 2022 Mar 7;11:giac015. doi: 10.1093/gigascience/giac015 (PMC8900497; doi:10.1093/gigascience/giac015)
Supplement: giac015_Reviewer_1_Report_Original_Submission — Alex Wiesman -- 11/16/2021 Reviewed [file giac015_reviewer_1_report_original_submission.pdf]

Reviewer Report

Title: The Nencki-Symfonia EEG/ERP dataset: Multiple cognitive tasks and resting-state data collected in a sample of healthy adults

Version: Original Submission **Date:** 11/16/2021

Reviewer name: Alex Wiesman

Reviewer Comments to Author:

The dataset described in this paper includes high-density (128 electrodes) EEG from 42 participants during their performance of 3 well-established cognitive tasks, as well as a resting-state protocol. The procedures and equipment used to collect the data appear to be of high quality and the data and auxiliary files made available are very thoroughly described. Overall, the paper is well written and these data will be very useful to researchers interested in using EEG/ERPs to study questions related to attention, executive function, and processing speed. I have only minor comments:

1. The second sentence of the "Background" section of the abstract is somewhat vague - I would recommend specifying further or removing.

In the same section there is a reference to a "resting-state task" - technically resting-state isn't a task.

2. On page 4, the descriptions of stimulus-stimulus and stimulus-response interference reference only visual/spatial forms, but these types of cognitive interference have been shown to extend to other sensory domains and to temporal processing.

3. Including a "Summary of the limitations of this data set" section (alongside the "advantages" section) might also be useful. Some options that could be included:

3a. The authors are correct that methodological differences hinder cross-study comparisons in this field, and that sharing this data set will help overcome these limitations. However, it does not overcome what is perhaps one of the most important differences: variability in imaging methodology (e.g., fMRI vs EEG vs MEG vs ECoG).

3b. The relatively short stimulus and ISI durations might not allow for thorough investigation of more sustained brain responses, e.g., of the effects of cognitive interference/conflict on oscillatory motor dynamics, which can last 1-2 seconds after movement termination and which our group and others have shown are potentially of-interest (Gaetz et al., 2013; Grent-'t-Jong et al., 2013; Heinrichs-Graham et al., 2018; Wiesman et al., 2020).

4. Can the "extended psychometrics" that are also available upon request be described briefly in the paper? Or better yet, can they be provided alongside these data?

5. Participant's highest level of education might be expected to covary with performance on these tasks, and particularly on the MSIT. Was this data collected and can it be supplied?

6. Can the Polish adaptation of the ARSQ also be shared alongside these data? I would imagine that this took a sizeable amount of work to put together, and would likely be very useful to many researchers.

7. More details regarding the task training would be useful. For example, were the participants allowed to practice as much as they wanted, or was there a time limit?

8. Why were the MSIT stimuli presented in "mini-blocks"? Does this align with previous studies in this

area (e.g., the original work by Bush et al.)?
9. On page 12, the line "…measure of the basic capabilities of the participants" might be more accurate as "…measure of the basic motor and processing speed capabilities of the participants".
10. Have any peer-reviewed manuscripts been published using these data? If so, it would be helpful to reference them as well (maybe on page 16) so readers can inspect the quality of the data further.
11. Can the average impedances be provided for the other tasks as well? I only see them for MSIT.
12. Were any statistics performed to support the conditional differences in the ERPs? This wouldn't be entirely necessary (as the general patterns are only being used to infer reliability), but might be useful to some readers.
13. It's unclear from Figures 2 and 3 (and from the methodological description) whether a blank screen or fixation point was presented during the ISI period for the MSIT and oddball tasks. Can this information be provided?
14. Plotting the individual data points in Figure 4 would be useful.

References:

Gaetz, W., Liu, C., Zhu, H., Bloy, L., Roberts, T.P., 2013. Evidence for a motor gamma-band network governing response interference. Neuroimage 74, 245-253.
Grent-'t-Jong, T., Oostenveld, R., Jensen, O., Medendorp, W.P., Praamstra, P., 2013. Oscillatory dynamics of response competition in human sensorimotor cortex. Neuroimage 83, 27-34.
Heinrichs-Graham, E., Hoburg, J.M., Wilson, T.W., 2018. The peak frequency of motor-related gamma oscillations is modulated by response competition. Neuroimage 165, 27-34.
Wiesman, A.I., Koshy, S.M., Heinrichs-Graham, E., Wilson, T.W., 2020. Beta and gamma oscillations index cognitive interference effects across a distributed motor network. Neuroimage 213, 116747.

Level of Interest

Please indicate how interesting you found the manuscript: Choose an item.

Quality of Written English

Please indicate the quality of language in the manuscript: Choose an item.

Declaration of Competing Interests

Please complete a declaration of competing interests, considering the following questions:

- Have you in the past five years received reimbursements, fees, funding, or salary from an organisation that may in any way gain or lose financially from the publication of this manuscript, either now or in the future?
- Do you hold any stocks or shares in an organisation that may in any way gain or lose financially from the publication of this manuscript, either now or in the future?
- Do you hold or are you currently applying for any patents relating to the content of the manuscript?

- Have you received reimbursements, fees, funding, or salary from an organization that holds or has applied for patents relating to the content of the manuscript?
- Do you have any other financial competing interests?
- Do you have any non-financial competing interests in relation to this paper?

If you can answer no to all of the above, write 'I declare that I have no competing interests' below. If your reply is yes to any, please give details below.

I declare that I have no competing interests.

I agree to the open peer review policy of the journal. I understand that my name will be included on my report to the authors and, if the manuscript is accepted for publication, my named report including any attachments I upload will be posted on the website along with the authors' responses. I agree for my report to be made available under an Open Access Creative Commons CC-BY license (http://creativecommons.org/licenses/by/4.0/). I understand that any comments which I do not wish to be included in my named report can be included as confidential comments to the editors, which will not be published.

Choose an item.

To further support our reviewers, we have joined with Publons, where you can gain additional credit to further highlight your hard work (see: https://publons.com/journal/530/gigascience). On publication of this paper, your review will be automatically added to Publons, you can then choose whether or not to claim your Publons credit. I understand this statement.

Yes Choose an item.
