# Supplementary material for: The Nencki-Symfonia electroencephalography/event-related potential dataset: Multiple cognitive tasks and resting-state data collected in a sample of healthy adults
Source: Gigascience. 2022 Mar 7;11:giac015. doi: 10.1093/gigascience/giac015 (PMC8900497; doi:10.1093/gigascience/giac015)
Supplement: giac015_Reviewer_2_Report_Original_Submission — Georgios Dimitrakopoulos -- 11/18/2021 Reviewed [file giac015_reviewer_2_report_original_submission.pdf]

Reviewer Report

Title: The Nencki-Symfonia EEG/ERP dataset: Multiple cognitive tasks and resting-state data collected in a sample of healthy adults

Version: Original Submission **Date:** 11/18/2021

Reviewer name: Georgios Dimitrakopoulos

Reviewer Comments to Author:

In this work an EEG experiment was performed, using several tasks (Multi-Source Interference Task, oddball, simple reaction task, rest state). The experiment is interesting, the data are large (number of subjects, number of electrodes, additional psychometric measures), hence this dataset can be a useful resource. I only have some minor comments that need to be addressed:
-For the ERP results, what pre-processing steps were performed?
-The oddball reaction time could be included in Fig. 4 (or maybe remove SRT reaction time and leave only various MIST conditions)
-Sessions were performed in two time slots. Is there a chance that participants in the afternoon session are more fatigued and have reduced performance?
-In Tab. 2, in some cases there are several runs, but one is recorded. Is this the last run?
-How many trials were on average in MIST task?
-When first referring to impedance, you mention Tab. 7, but at this point it should be Tab. 2. Moreover Tab. 7 and 8 are mentioned, but there is no Tab. 4-6.

Level of Interest

Please indicate how interesting you found the manuscript: Choose an item.

Quality of Written English

Please indicate the quality of language in the manuscript: Choose an item.

Declaration of Competing Interests

Please complete a declaration of competing interests, considering the following questions:

- Have you in the past five years received reimbursements, fees, funding, or salary from an organisation that may in any way gain or lose financially from the publication of this manuscript, either now or in the future?
- Do you hold any stocks or shares in an organisation that may in any way gain or lose financially from the publication of this manuscript, either now or in the future?

- Do you hold or are you currently applying for any patents relating to the content of the manuscript?
- Have you received reimbursements, fees, funding, or salary from an organization that holds or has applied for patents relating to the content of the manuscript?
- Do you have any other financial competing interests?
- Do you have any non-financial competing interests in relation to this paper?

If you can answer no to all of the above, write 'I declare that I have no competing interests' below. If your reply is yes to any, please give details below.

I declare that I have no competing interests

I agree to the open peer review policy of the journal. I understand that my name will be included on my report to the authors and, if the manuscript is accepted for publication, my named report including any attachments I upload will be posted on the website along with the authors' responses. I agree for my report to be made available under an Open Access Creative Commons CC-BY license (http://creativecommons.org/licenses/by/4.0/). I understand that any comments which I do not wish to be included in my named report can be included as confidential comments to the editors, which will not be published.

Choose an item.

To further support our reviewers, we have joined with Publons, where you can gain additional credit to further highlight your hard work (see: https://publons.com/journal/530/gigascience). On publication of this paper, your review will be automatically added to Publons, you can then choose whether or not to claim your Publons credit. I understand this statement.

Yes Choose an item.
